# Improving health information systems during an emergency: lessons and recommendations from an Ebola treatment centre in Sierra Leone

**DOI:** 10.1186/s12911-019-0817-9

**Published:** 2019-05-27

**Authors:** Shefali Oza, Kevin Wing, Alieu Amara Sesay, Sabah Boufkhed, Catherine Houlihan, Lahai Vandi, Sahr Charles Sebba, Catherine R. McGowan, Rachael Cummings, Francesco Checchi

**Affiliations:** 1 London School of Hygiene and Tropical Medicine, Keppel Street, London, WC1E 7HT UK; 2 Save the Children International, Kerry Town, Rural District, Western Area Sierra Leone; 3 University College London, Gower Street, London, WC1E 6BT UK; 4 Save the Children UK, London, 1 St John’s Lane, London, EC1M 4AR UK

**Keywords:** Ebola virus disease, Disease outbreaks, Health emergencies, Health information systems, Medical records, Health records, Electronic health records, Data collection

## Abstract

**Background:**

The 2014–2016 West Africa Ebola epidemic highlighted the difficulty of collecting patient information during emergencies, especially in highly infectious environments. Health information systems (HISs) appropriate for such settings were lacking prior to this outbreak. Here we describe our development and implementation of paper and electronic HISs at the Sierra Leone Kerry Town Ebola treatment centre (ETC) from 2014 to 2015. We share our approach, experiences, and recommendations for future health emergencies.

**Methods:**

We developed eight fact-finding questions about data-related needs, priorities, and restrictions at the ETC (“inputs”) to inform eight structural decisions (“outputs”) across six core HIS components. Semi-structured interviews about the “inputs” were then conducted with HIS stakeholders, chosen based on their teams’ involvement in ETC HIS-related activities. Their responses were used to formulate the “output” results to guide the HIS design. We implemented the HIS using an Agile approach, monitored system usage, and developed a structured questionnaire on user experiences and opinions.

**Results:**

Some key “input” responses were: 1) data needs for priorities (patient care, mandatory reporting); 2) challenges around infection control, limited equipment, and staff clinical/language proficiencies; 3) patient/clinical flows; and 4) weak points from staff turnover, infection control, and changing protocols. Key outputs included: 1) determining essential data, 2) data tool design decisions (e.g. large font sizes, checkboxes/buttons), 3) data communication methods (e.g. radio, “collective memory”), 4) error reduction methods (e.g. check digits, pre-written wristbands), and 5) data storage options (e.g. encrypted files, accessible folders). Implementation involved building data collection tools (e.g. 13 forms), preparing the systems (e.g. supplies), training staff, and maintenance (e.g. removing old forms). Most patients had basic (100%, *n* = 456/456), drug (96.9%, *n* = 442/456), and additional clinical/epidemiological (98.9%, *n* = 451/456) data stored. The questionnaire responses highlighted the importance of usability and simplicity in the HIS.

**Conclusions:**

HISs during emergencies are often ad-hoc and disjointed, but systematic design and implementation can lead to high-quality systems focused on efficiency and ease of use. Many of the processes used and lessons learned from our work are generalizable to other health emergencies. Improvements should be started now to have rapidly adaptable and deployable HISs ready for the next health emergency.

**Electronic supplementary material:**

The online version of this article (10.1186/s12911-019-0817-9) contains supplementary material, which is available to authorized users.

## Background

The 2014–2016 Ebola epidemic in West Africa was unprecedented not only in its scope and scale [1], but also in the challenges and opportunities it presented for collecting patient health records. In an emergency, health information systems (HISs) are needed to support patient care and to help coordinate the overall response. Rapidly implemented basic health records that “get the job done” are often justifiably prioritized over more comprehensive, high-quality data collection systems.

There are, however, several benefits to having a well-functioning HIS during a large-scale outbreak. First, such a system makes patient care more efficient and accurate [2]. Second, for diseases with a scant evidence base such as Ebola, patient data are essential for clinical trials and understanding prognostic factors for survival [3]. Even basic questions, such as the importance of intravenous (IV) fluids for survival, were hotly debated during this outbreak [4]. Patient records of adequate quality can help answer some of these questions. Third, easily producible patient data summaries are needed for staff and resource management, as well as for external monitoring and surveillance.

### HIS challenges for Ebola

The lack of established models for designing and implementing HISs for a large-scale Ebola outbreak was understandable since earlier Ebola outbreaks had fewer than 500 cases [5]. In comparison, this outbreak had more than 28,000 reported cases [6]. Pre-existing standardized data collection tools, like the Centers for Disease Control’s Epi Info viral hemorrhagic fever application [7], were intended for outbreak tracking rather than individual patient records. Other tools, like ISARIC’s initial case record form [8], were too detailed and research-focused to be directly useable in the Ebola treatment centre (ETC) environment. Moreover, no “system” existed that brought most or all of the necessary HIS components together cohesively.

**Fig. 1:**
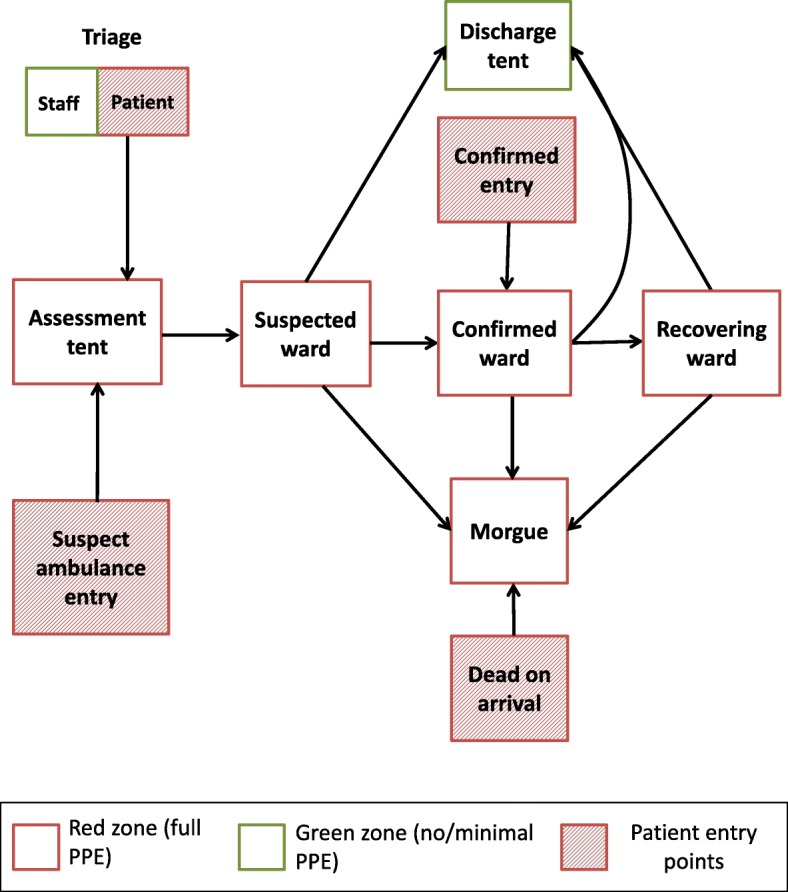
Schematic of people and equipment flow in the red zone of the Kerry Town Ebola treatment center

Recording of and communicating patient data in an ETC has particular challenges, largely due to severe infection control requirements [9]. The amount and quality of patient data that can be reasonably recorded is restricted by 1) personal protective equipment (PPE), which limits dexterity, visibility, and time with patients; 2) the inability to move paper patient records from highly infectious patient areas (red zone) to low risk areas (green zone); and 3) one-way movement of people and equipment from suspect to confirmed patient wards (Fig. 1).

These restrictions are in addition to the more general challenges presented during emergencies (especially in low-resource settings), such as high staff turnover, varying skill/experience levels of clinical staff, multiple languages amongst clinicians and patients, limited data collection training, fast-paced changes to treatment protocols, and critical but time-consuming reporting to external actors.

### Paper versus electronic health records

Health records may be paper-based, electronic, or a combination of the two. Paper health records (PHRs) are familiar, easier to develop and modify, and generally inexpensive. However, PHRs can be damaged or lost, hard to use with PPE, and more error-prone. Version control, especially of already circulated forms, can also be difficult. Electronic health records (EHRs) can result in higher-quality/standardized data entry, be configured for efficiency and review in difficult environments, and have automated functions for reporting and quality checks [10]. But they can require expensive/fragile equipment, more time and programmers to develop, and additional training. In the ETC, EHRs have a critical advantage in transmitting information between the red and green zones. While paper records cannot physically leave the red zone, electronic records can be instantaneously communicated over a wireless local area network (WLAN) even in settings with unreliable power and no Internet connection.

### Goals of this paper

The HIS for the Kerry Town ETC in Sierra Leone, operated by Save the Children International (SCI), consisted of both paper and electronic health records. This paper describes the system-level processes we used to develop and implement the overall HIS structure at this site, including integration of the HIS within the wider ETC environment. A previous paper [11] describes technical details of the EHR development and implementation, so we will not go into depth on those topics in this paper. In contrast, our goal here is to describe the higher-level strategic approach we used, discuss lessons learned, and provide recommendations for how high-quality patient health records can be more efficiently collected in future outbreaks and emergencies.

## Methods

### Setting

SCI operated the 80-bed Kerry Town ETC from November 2014 to March 2015. This multi-building facility had distinct red and green zones. The red zone comprised patient areas such as triage and wards for confirmed, suspect, and recovery patients. The green zone had a clinicians’ station, pharmacy, warehouse, kitchen, and offices for the health, sanitation, operations, patient care, and HIS teams (Additional file 1; section A1). There were two on-site laboratories. The over 100 clinical staff, ranging from nursing assistants to doctors, were a mix of long-term Sierra Leoneans, medium-term Cuban volunteers, and some additional short-term internationals. Common languages amongst the clinical staff included Krio, English, and Spanish, with most of the international staff speaking only English or Spanish. Patients spoke mostly Krio or other local languages.

### Review of existing data collection solutions

Prior to designing our patient records, we searched for possible PHR and EHR solutions that could be quickly adapted for our setting. We did this by: 1) searching academic literature (using Google Scholar and PubMed) and non-academic documentation (e.g. through Google), 2) directly approaching organizations involved in opening large-scale ETCs in West Africa, and 3) having informal discussions with individuals experienced in data collection during emergencies (especially for Ebola or other outbreaks). Because of the rapid pace required, these searches were conducted over the scale of hours to days, with some discussions continuing for a few weeks. Our academic literature search included disease search terms of “Ebola” or “viral hemorrhagic fever” and data-related search terms of “data collection”, “medical records”, “patient records”, “patient data”, “documentation”, “health information system”, and “health information”.

### Determining inputs and outputs for the HIS

To design the Kerry Town ETC HIS content and structure, we first identified the six core components that we believed were necessary and feasible for a comprehensive HIS at our site (Table 1). This list was developed through informal discussions within our team based on our experiences with data collection systems in emergency and non-emergency situations.

**Table 1:** Core components of a health information system during an emergency

Core HIS component	Description
Data collection tools	Paper and/or electronic forms that constitute the information (e.g. demographic, epidemiological, medical) collected on individual patients.
Communication of data	Methods and channels for communicating patient information as securely as possible to relevant parties within the care facility, and externally as necessary.
Coordination amongst relevant parties	Channels for up-to-date communication with the individuals and departments that play a role in using and/or maintaining the HIS.
Staff training	Development and use of standardized training tools, schedules, and sessions related to HIS, including medical ethics.
Data management	All aspects of managing data, including data digitization, entry, storage, security, and archiving.
Data analysis and reporting	Processing and analyzing data for monitoring care, internal/external reporting, and research.

We then mapped out a set of fact-finding questions about patient data needs and capabilities/resources at the ETC (“inputs”) to inform structural decisions (“outputs”) for the HIS across these six core components (Table 2). A similar informal team-based discussion approach was used to develop and revise these inputs and outputs.

**Table 2:** Inputs and outputs for designing a health information system during an emergency

Inputs from key stakeholders	
I1	What are the priorities for the use of the patient data?
I2	What patient data are needed to meet these priorities?
I3	What challenges may restrict data collection?
I4	What is the patient flow at the site?
I5	What is the clinical workflow at the site?
I6	Who needs access to which data, where, and how often?
I7	Which activities may interfere with the HIS?
I8	What resources (equipment, money, personnel, infrastructure, time) are available for building and using the system?
Outputs to guide design of the health information system	
O1	Which data and where to collect them
O2	Platform for data collection
O3	Design decisions for data collection tools
O4	Who collects the patient data
O5	How data are communicated to the necessary people
O6	How data errors are minimized
O7	How the patient records are digitized and processed
O8	How and where the patient records are stored and secured

We then selected stakeholders to answer the input questions. We chose these stakeholders by reviewing HIS activities/needs in each ETC department and amongst external partners. For any teams with data or maintenance linkages to the HIS, the team leaders were chosen as stakeholders. For those strongly connected to the HIS (e.g. clinical team), 1–3 non-leader staff members were also included to represent different user perspectives. The stakeholders included clinicians, epidemiologists, pharmacists, lab specialists, information technology (IT) personnel, water/sanitation/hygiene (WASH) staff, ETC operations management, and various internal teams and external partners that needed specific or aggregate patient data. We used informal semi-structured interviews to ask each stakeholder the input questions relevant to their team. We then used their responses to formulate the output decisions, framed in the context of strict infection control, through discussions within our team. Time constraints ruled out a more formal approach. The inputs and outputs were part of an iterative process, with answers changing based on evolving needs as well as trial and error during implementation.

From the outset, we chose to develop both a PHR system (to be ready for the ETC opening) and an EHR system (to address the critical red zone communication challenges, but longer to develop) (Additional file 1, section A2). Most of the inputs/outputs were for higher-level structural decisions, and thus applicable to both the PHR and EHR systems. For areas where the two systems diverged, we sought inputs and formulated outputs for each system.

### Implementing the HIS

The key tasks for implementing the HIS included: 1) building the PHR forms/databases and EHR software, 2) preparing the systems for use, 3) training staff on using the systems, 4) further revisions based on feedback and monitoring, and 5) maintaining the systems.

### System usage and evaluation

PHR and EHR system usage was monitored through both routine clinical care and retrospective analyses of patient records. We assessed missing individual records across different aspects of patient care and tried to identify why the records were missing.

Additionally, we developed a structured questionnaire on clinicians’ experiences with the PHR system and opinions on an EHR system as part of routine feedback for system improvements (Additional file 1, section A3). This questionnaire was available in English and Spanish. Clinicians were asked (through team leaders, word-of-mouth, and in person) to complete it in February 2015, three months after the ETC opened and prior to the implementation of the EHR system. We also documented informal feedback received from HIS users through the duration of ETC operations. A comparison of patient data that were entered in both the PHR and EHR systems is described elsewhere [11].

## Results

### Review of existing data collection solutions

We found no publication with guidance on designing an overall HIS for Ebola or other viral hemorrhagic fever (VHF) outbreaks. Buhler et al. discussed red to green zone communication methods [12], and some previous clinical publications noted the need for quality data collection [13, 14]. Most of the organizations we contacted had not yet set up data collection systems for the Ebola outbreak and none had one that suited our needs. Médecins Sans Frontières (MSF) gave us valuable advice on data collection and communication challenges. Through informal discussions, we learned about the ISARIC case record form and CDC’s case investigation form. Based on these discussions and our previous experiences, we were aware that most data collection during emergency outbreaks was minimal. See section A4 of “Additional file 1” for details on our EHR platform search.

### Determining inputs and outputs for the HIS

Table 3 summarizes our findings from the stakeholder interviews (inputs) and our HIS decisions based on their responses (outputs).

**Table 3:** Summary of input and output results for designing the Kerry Town ETC health information system

Brief description of inputs/outputs^a^	Summary of results
Inputs	
I1: Data use priorities	High priority: patient care; mandatory reporting to the government and SCI leadership; Medium priority: longer-term medical research; Low priority: assessing data collection quality control, actively informing treatment practices.
I2: Necessary patient data	Patient care: baseline (intake) information and daily medical records; Reporting: daily status updates by various breakdowns (e.g. demographics, outcome); Research: detailed medical records, epidemiological factors.
I3: Data collection challenges	Data collection restricted by PPE, limited ETC equipment/facilities, high variance in clinical skill levels, language differences between patients/staff and amongst staff.
I4: Patient flow	See Fig. 1.
I5: Clinical workflow	Strictly designated rotations for patient rounds, medication administration, food, and other care during morning, afternoon, evening, and night shifts. Patient data were collected and reviewed in the red and green zones depending on the task. Similar to patients, clinicians also had one-directional flow in ETC. See section A5 of Additional file 1 for more specific details.
I6: Data access – who, which, where, how often	Red zone: bedside patient records for clinician rounds; green zone: daily individual patient data for clinical, pharmacy, laboratory, HIS, and patient care (e.g. psychosocial and community) staff; aggregate daily patient numbers for operations management team; weekly aggregate updates for leadership.
I7: Activities interfering with HIS	WASH staff incinerating red zone materials could destroy patient records; clinical protocol changes could alter HIS workflow; staff turnover and limited handover time could affect system maintenance; infection control means monitoring system is difficult in red zone.
I8: Available resources	Equipment: generators, printer/copier, scanners, laptops, office supplies (toner, paper). Additional for EHR - waterproof tablets, server, Wi-Fi routers with uninterruptable power sources (UPSs). Money: minimal needed for PHR, additional £25 k pounds from SCI for EHR development/equipment. Personnel: 1–5 person site-based HIS staff and 1–2 overseas advisors for PHR; additional off-site software development team for EHR [11]. Time: requirement for functional (but later modifiable) HIS needed for site opening.
Outputs	
O1: Which data and where	We categorized possible patient data as essential versus desirable, aiming to start with essential only. We mapped data flow across the ETC including how to split data across forms/modules and rooms (Additional file 1; section A6).
O2: Data platform	We used 1) Microsoft Word to adapt ISARIC’s case record form and develop other forms for the PHR, 2) EpiInfo and EpiData databases for some PHR data entry, and 3) OpenMRS for the EHR. We converted final paper forms to PDFs.
O3: Data collection design decisions	For the forms/modules, we aimed for minimal information, large font sizes (no smaller than size 14 on forms) for entry/review in red zone, checkboxes or buttons for faster and clearer entry/review, ordering information in intuitive ways (e.g. symptoms from top to bottom of body). Examples of these decisions are demonstrated in the PHR baseline assessment form (Fig. 2) and the EHR IV fluid ordering/monitoring module (Fig. 3).
O4: Who collects data	Selecting which staff should record patient data varied based on which data were being collected, data collection platform being used, preferences of medical lead, and staff member availability and skills (e.g. language, computing, writing).
O5: How data are communicated	Site layout and infection control dictated communication methods. Key approaches were 1) radio transmission (typically for complicated information i.e. drug orders, vital signs), 2) “duplicate” charts in green zone from “collective memory” of clinicians returning from red zone (typically general information i.e. patient status, symptoms), and 3) red zone Wi-Fi scanner (only retrospectively due to logistical problems). Clinicians brought in patient record copies from green zone to review in red zone. EHR automatically transmitted information over the Wi-Fi router to on-site server for access by Wifi-enabled devices.
O6: How errors are minimized	We 1) assigned patient ID numbers with a check digit (instead of sequential) final number (Additional file 1; section A7), 2) carefully handwrote patient wristbands in advance, 3) reviewed log books when individuals patient files had errors or unusual values, 4) performed retrospective accuracy checks on subset of digitized data and data re-entry.
O7: How records are digitized / processed	HIS staff used simple EpiData database to digitize data needed for daily and weekly reporting from PHR forms. De-identified data were exported and converted into Stata format for analysis and report production. Additional patient data were retrospectively digitized by securely scanning PHR forms and entering selected data into EpiInfo database. EHR exported data into a CSV file.
O8: How and where records are stored/secured	Patient records were stored in: 1) red zone until patient visit was over; 2) green zone clinicians’ station for log books and duplicate charts of current patients, and 3) longer-term storage in a locked HIS office cabinet for discharged patient files. Scans and databases were secured using Safehouse Explorer Encryption software on password-protected laptops stored in a locked cupboard. Some departments stored own additional patient records, including pharmacy and labs. EHR data were on a secure green zone server with nightly backups. Clinical staff logged in to access patient files but only HIS team lead could access downloadable files on server.

^a^ See Table 2 in the methods sections for a full description of the inputs and outputs

**Fig. 2:**
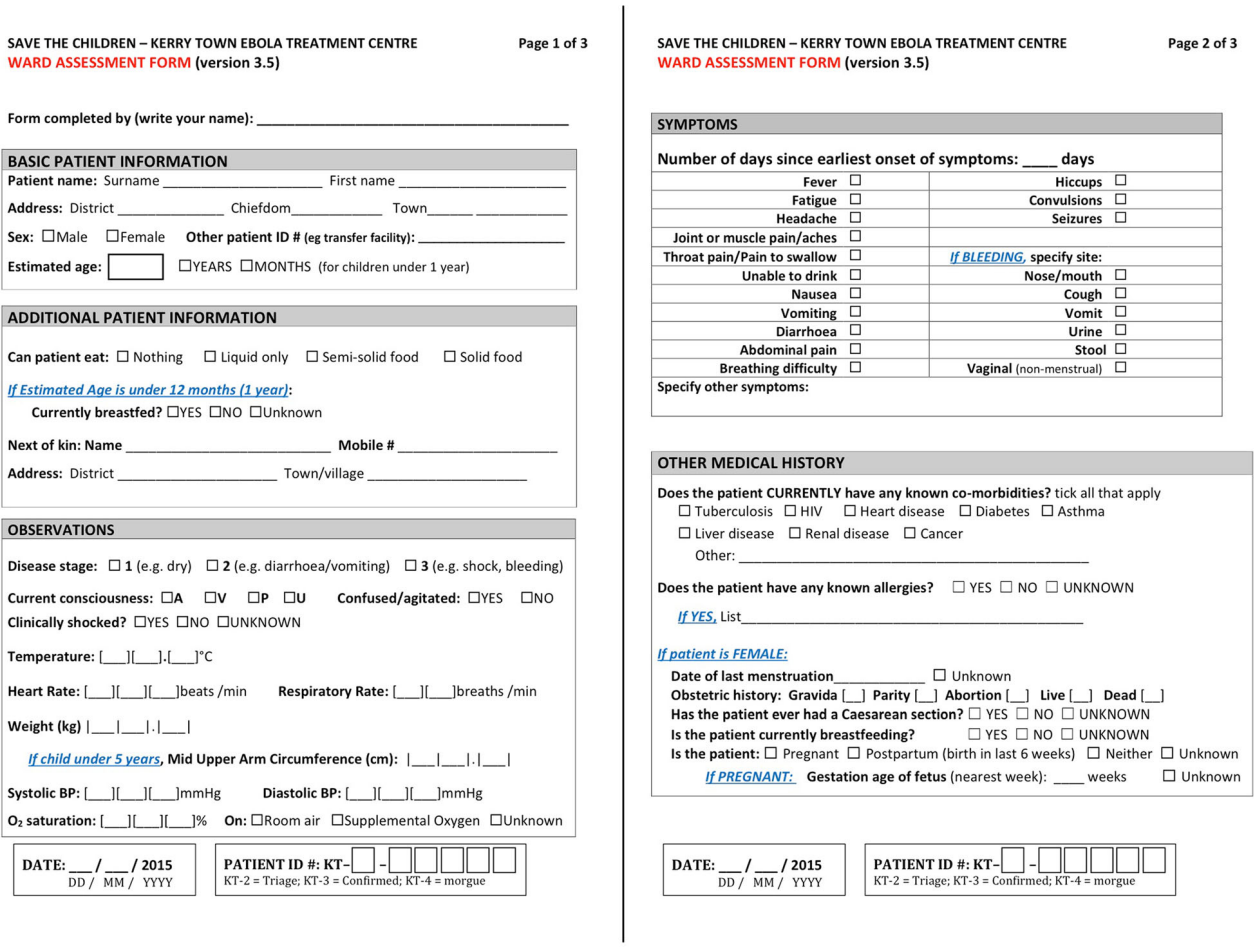
The first two pages of the baseline ward assessment paper form

**Fig. 3:**
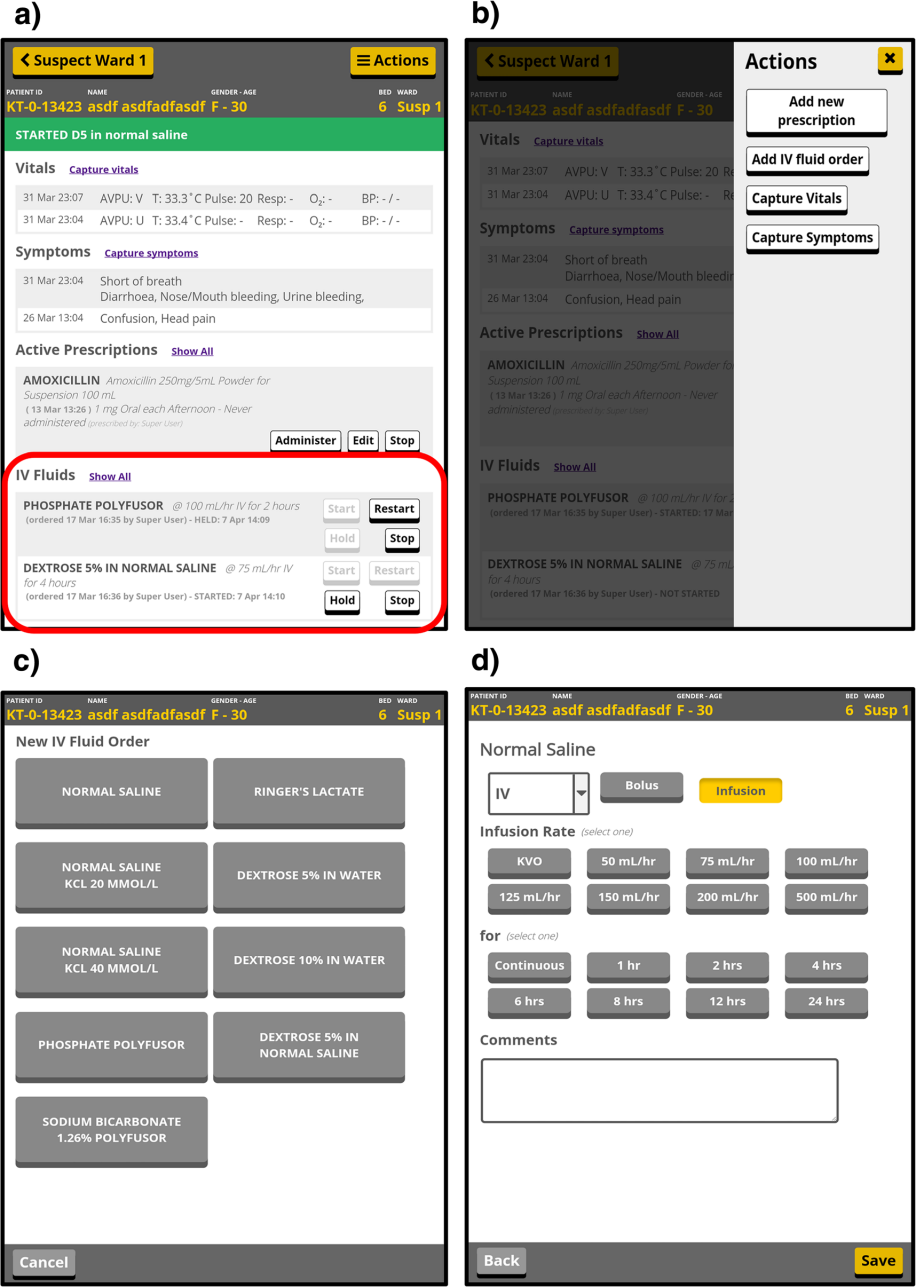
Screenshots of the intravenous fluid (IV) ordering and monitoring module of OpenMRS-Ebola. Legend: Patient summary screen with current IV fluid orders (with start, hold, restart, and stop buttons) (**a**), screen that opens with a button for adding new IV fluids when action button on patient summary screen is pressed (**b**), nine IV fluids available for ordering (**c**), and screen with buttons to select infusion rate and time period for the selected IV fluid (**d**)

### Implementing the HIS

#### Data collection forms/modules

We made 13 forms for the PHR system, had two log books for the clinicians’ station, and retained case investigation and intake forms for patients from their previous Ebola holding centres. Essential forms were implemented on the day the site opened. Additional forms, including IV fluid infusion charts, nutrition forms, and a patient exit survey, were added based on staff feedback. A triage form was deployed when the suspect ward opened. See section A6 of “Additional file 1” for PHR form descriptions and rollout dates and “Additional file 2” for copies of the PHR forms. The PHR databases were built and implemented over the first two months. We made eleven key modules for the EHR system, with five for the tablet-based application and six for the desktop application [11].

#### Preparing the systems

For the PHR system, we ordered necessary HIS supplies; bulk printed forms; prepared pre-written wristbands; establishing dedicated physical spaces for blank forms and in-use patient record folders in the clinicians’ station and wards; identified storage space in the HIS office for completed records; and, more generally, ascertained that the “outputs” were completed. Additional EHR tasks, like deploying tablets and field testing, are described elsewhere [11].

#### Staff training

Clinicians were trained on how to use the PHR system using Powerpoint presentations. We trained and obtained feedback from initial clinical staff prior to the ETC opening. Later incoming staff received HIS training upon arrival as part of their introductory clinical training. The training included an overview of what HIS entails and how it fits into the ETC, a description of each PHR form, “do”s and “don’t”s of properly filling out forms, and planned improvements. Printed forms were provided to clinicians for familiarization. Any further HIS trainings (e.g. refresher sessions) were conducted over several days while clinicians were on break between shifts. EHR training is described elsewhere [11].

#### Revisions

We made several system modifications based on user feedback, especially during the first two months of the site opening. The majority of revisions were focused on ease of use, feasibility, and usefulness. The key changes to the forms were to: 1) add forms based on clinical feedback (e.g. IV fluid charts, nutritional intake); 2) add requested questions and delete unnecessary ones (e.g. vitals or signs not collected in practice due to time constraints); 3) increase font sizes for red zone forms; and 4) move item locations on forms to improve intuitive flow. We also attempted several red to green zone communication methods, including ones that were too time consuming or difficult to implement. See “Additional file 1, section A8” for more details on various revisions we made to HIS components.

#### System maintenance

This included tasks such as ensuring that only new versions of forms were in circulation, staff turnover did not disrupt the system, clinical protocol changes were incorporated into the HIS structure, and generally that the various HIS components were operating well.

### System usage and evaluation

**Fig. 4:**
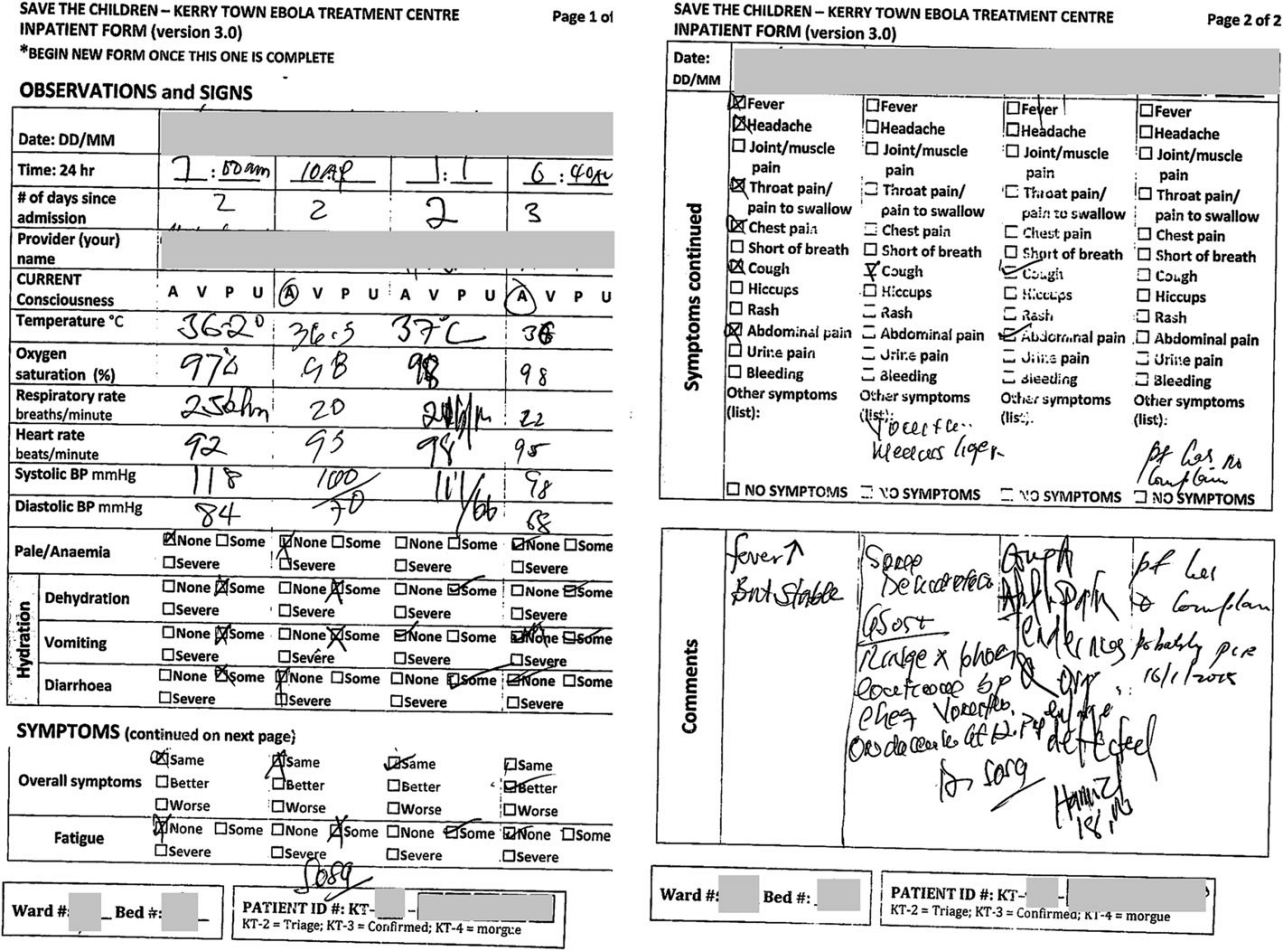
An inpatient form completed in the infectious (red) zone at the Kerry Town Ebola treatment center

The PHR system was used for all 456 suspect and confirmed Ebola patients admitted to the Kerry Town ETC. Basic demographic, Ebola status, and outcome information was recorded for all patients. For 451 patients (98.9%), additional epidemiological, clinical, and/or administrative data were collected and stored. Medication recordings were available in the drug charts of 96.9% (*n* = 442) of patients. On average, about 2–3 pages were added to the patient file for each additional day at the ETC. Red zone records were retained for 40% (105/264) of Ebola-positive patients (excluding five who were dead on arrival). Lack of red zone records were due to being 1) lost or damaged in the red zone, 2) incinerated prior to scanning, or 3) not collected in the red zone. An example of an inpatient form completed in the red zone and scanned retrospectively is shown in Fig. 4. See section A9 of “Additional file 1” for other results, including on missing data. EHR usage details are published elsewhere [11].

Sixteen (of approximately 70) clinical staff, including community health assistants and officers, nurses, and doctors, completed a structured evaluation questionnaire in February 2015. These clinicians included five Sierra Leoneans, six Cubans, and five from other countries. We had difficulty with further participation because of clinician availability and exhaustion between shifts. PHR aspects the clinicians liked included the checkboxes on the forms; ability to document clinical information throughout the patient stay; better handovers because of documentation; simplified forms; and ability to review charts inside and outside of wards. Common responses about PHR challenges included difficulty collecting and viewing records while wearing PPE in the red zone; radio working well for communicating information in wards but disturbing patients; limited time in red zone to record information after patient care, easily damaged and sometimes misplaced records in the red zone; many clinicians using each patient’s record; and the challenge of memorizing information for duplicate green zone records. On several data collection topics, opinions were divided. Six respondents preferred data collection in the red zone because relying on radio and memory for green zone records was difficult, while eight strongly preferred green zone recording only. Ten of those surveyed preferred having patient records available in the red zone, but three thought this was not useful. Opinions on the drug and IV fluid charts were the most varied, with about half of questionnaire respondents saying the process worked well while the rest found it to be the most challenging aspect of the PHR system. This is unsurprising since accurate drug ordering is essential; this particular challenge was a key factor when we considered building an EHR system. Nearly all (15/16) believed an EHR could improve patient record collection because of the challenges of using paper in the red zone [11].

## Discussion

We built and implemented paper-based and electronic data collection systems for patient records at the Kerry Town Ebola treatment centre in Sierra Leone. Both the quality and quantity of patient data varied over time based on staff training, clinical leadership, protocol changes, and the methods used to communicate information from the red to green zone. Here, we discuss the HIS challenges we faced and present recommendations based on our experiences and lessons learned.

### General design and implementation lessons

In the time-limited and often chaotic environment of an emergency, ad-hoc creation and amalgamation of HIS components is more common than planning a well-designed system. Typically, data collection tools will be created with limited to no design processes or user feedback, minimal formal training, and extempore revisions. If paper-based data are digitized, data entry is usually done using spreadsheets rather than databases. This more ad-hoc approach is understandable given the urgency of an emergency setting. However, a comprehensive HIS can be relatively quick to design and is likely to save substantial time later, improve data quality, and facilitate ongoing system maintenance. We found that using Tables 1 and 2 together – mapping out the inputs and outputs to design processes across the six core HIS components – helped us strive for a more systematic approach.

First, the best way to design a successful *complex* health record system in an emergency is to begin with a *basic* one that captures only essential patient information. After sufficient training and data quality evaluation, more complexity can be added. This allows users with different proficiencies (including language) to become comfortable entering the basic set of data, and provides HIS staff an opportunity to evaluate implementation problems and alter training as needed. One difficulty with implementing a basic system is that requests to add or remove questions are common. Competing priorities amongst various individuals and departments resulted in our own initial forms having more questions than the minimum needed for care, and this became a key challenge for system rollout. While the approach of starting with basic functionality may initially cause friction, we believe it is the optimal approach for obtaining high-quality data in settings with many users, little training time, and diverse skillsets/backgrounds.

We used an Agile development approach [15] for both the PHR and EHR systems. This was particularly useful for quick implementation during an emergency, but required regular attention to find a balance between making necessary changes and maintaining a system on which users were already trained. For us, the two most challenging areas were 1) ensuring paper and EHR data capture were appropriate for the setting and users and 2) communicating information from the red to green zone. The latter, which was primarily a problem for the PHR system, required the most trial and error.

### Planning for high-quality data collection

Five important ways to maximize high-quality data collection are 1) designing simple, intuitive, easy-to-use forms (as discussed above), 2) user training and re-training as needed, 3) regularly monitoring system usage, 4) incorporating simple error-reduction techniques, and 5) ensuring sufficient HIS staffing. These are not easy to accomplish, and we struggled with several of these. But they are doable, and if planned from the start, can help maintain smooth operation of an HIS during high turnover of a diverse staff.

For training, simple tools can include individual copies of forms for review and practice, laminated examples of completed/annotated forms in common areas, and slide decks demonstrating good and bad data collection practices. Having standardized training tools ready, along with a plan for when and how refresher training will be done is important. Whether all clinicians or a small group of “superusers” will record data affects both training decisions and clinical workflow. Although selecting “superusers” can result in higher data quality, this approach increases overall process complexity (e.g. contingencies needed for staff absences or changes).

Ongoing system monitoring can then be performed, ranging from retroactively reviewing data collection forms and modifying trainings accordingly to conducting user surveys and interviews. Incorporating monitoring into the HIS from the start is optimal. For instance, having staff record their names, initials, or ID numbers on records can help during retroactive review and refresher training. EHRs can be programmed to facilitate monitoring, including by easily identifying users and automating data quality checks.

Examples of techniques to reduce long-term system errors include maintaining updated version numbers and dates on forms, use of pre-written wristbands and sticker labels, and using non-sequential patient ID numbers. Patient ID number mix-ups, which are particularly common with handwritten data, can have serious consequences. To mitigate this problem, we believe that the use of check digits should be the norm for patient ID numbers. At our site, we experienced several problems with ID numbers but were able to resolve these mostly because we used check digits. Other error reduction approaches include attaching stickers with patient ID numbers to each patient record page, double entry of patient data during digitization, and scannable radio-frequency identification (RFID) tags on wristbands and stickers if feasible.

With five HIS staff performing activities across the six core HIS components (Table 1), we were able to oversee the system and maintain daily operations. These staff included an on-site team leader to develop and oversee the system and liaise with other leadership, a manager to oversee daily HIS tasks, and three data entry clerks to digitize records needed for immediate and anticipated uses. Additional staff would have been useful for better monitoring of the system, double data entry, and further digitization of paper records (which we were unable to accomplish until well after the ETC closed). Remote assistance with some minor tasks could have also saved valuable time.

### Connections between the HIS and other teams

At our ETC, HIS users included HIS, clinical, pharmacy, laboratory, WASH, IT, patient care, management, and logistics staff. This diffuse network meant that several potential weak points existed in our system. Important information could be missed due to the high staff turnover and rapid changes to protocols and operating procedures. For example, valuable patient data stored in the red zone was mistakenly incinerated, likely due to a lack of communication between the HIS, clinical, and WASH teams during a period of high staff turnover. Such problems could be mitigated by having 1) an HIS-led organizational chart detailing which departments are responsible for which HIS system tasks, 2) written HIS protocols and training with the different departments, 3) regular communication with all relevant departments, and 4) detailed handover plans. Importantly, these strategies need to be followed at the beginning and throughout the emergency to help prevent problems that are much harder and time-consuming to fix later.

A strong link between the HIS and clinical teams is paramount for a smoothly operating HIS. Disruptions within and between these teams are common during an emergency. For example, new teams often want to change forms or communication methods. Although this could yield improvements, it can also wreak havoc on well-established systems on which other users are already trained. We frequently experienced this tension at our site. Ways to mitigate such disruption include having strong communication between the HIS and clinical leads; prioritizing minimal turnover in HIS and clinical leadership positions; involving long-term clinicians (who are often local rather than international staff) in HIS decisions and training; preparing for handovers with detailed written notes; documenting system design decisions in a shareable format; presenting key statistics regularly (e.g. weekly) to demonstrate the immediate value of a good HIS to staff; and incorporating design decisions into HIS user training to provide context.

### Ethical concerns

During emergency situations, confidentiality may appear to be a luxury compared to the difficult task of providing rapid patient care. However, ethical failings can have repercussions for the individual patient and the health system (especially for a disease as stigmatized as Ebola). Patient confidentiality violations can range from minor to serious. For example, shouting, publicly visible white boards, and radio communication were common mechanisms to convey patient information from the red zone during this outbreak. For ease of access to patient records, our own patient files were in an unlocked clinicians’ station during the patient’s stay, and were only locked away after their discharge or death. We also used a whiteboard there which initially included patient names (subsequently changed to ID numbers only). An EHR system has an inherent advantage here because it can be secured with a password, have restricted access, and accesses can be logged with usernames and timestamps.

For clinicians, an emergency response is often different from their normal clinical environment, so an ethics refresher focused on this emergency context is highly advisable. Additionally, non-clinical staff in such emergencies may know information about patients, but have no formal confidentiality training. At our site, the majority of our 600+ staff were non-clinical, and many of the patients came from the same communities as the local staff. Approaches for handling patient confidentiality (with HIS staff well-placed to coordinate) include: 1) enacting a policy on confidentiality (and violation consequences), 2) training all staff and clearly communicating the organizational policy, 3) having staff members sign a document committing them to confidentiality, and 4) enforcing the policy through further training, warnings, and dismissals if necessary. The WHO has developed a useful training document with additional advice on patient confidentiality and other ethical issues encountered during health emergencies [16].

Finally, precautions need to be taken to ensure the ethical use of patient data for research, particularly during emergencies with high research value (e.g. emerging or ill-understood diseases such as Ebola). Ethical review of research proposals is mandatory, and organizations like MSF and WHO have made important contributions towards this [17–19]. An internal process is needed within organizations to streamline and manage clinician research requests, and to prevent inappropriate use of clinical data. Our process involved channeling all research requests to two designated staff members for initial review prior to further ethical clearance. Clearly and regularly communicating this process to clinicians is critical, especially with high staff turnover.

### Sharing across organizations

Proactively sharing any available HIS components with other organizations can help save substantial amounts of time for others; create informal standardization that can aid with system compatibility and data comparability; and improve one’s own systems, whether through feedback on shared systems or others more willingly sharing their own systems. Furthermore, promoting a culture of openness and sharing is an important goal in itself, and should be the norm. Although the public health community has acknowledged that sharing data during an emergency is essential [20, 21], similar considerations have not been easily applied to HIS components. Common reasons not to share include: 1) that sharing takes additional time in a very time-limited setting, 2) not wanting to release preliminary forms or focusing on other priorities by the time the forms are finalized, 3) there being no common easy-to-use platform for sharing, and 4) it not yet being normal practice.

But successful examples demonstrating the benefits of sharing HIS components exist. In this outbreak, MSF shared training, tools, and protocols with others [22]. We circulated our preliminary paper-based forms over email, in person, and on the OpenMRS wiki [23]. We know of at least two organizations that adapted our forms for their ETCs: International Medical Corps [24] and Partners in Health (E. Ball, written communication, January 2015). We also directly shared our experiences, including mistakes, with HIS teams from newly opening ETCs during the outbreak. For the EHR, we intentionally chose an open-source platform to facilitate sharing. MSF’s Project Buendia also used the OpenMRS platform as its backend and we were able to share clinical vocabulary across the projects [25].

Potential ways to make sharing easier and more common include: 1) encouraging a culture of sharing resources (including preliminary versions), 2) having an easy-to-use open-source platform to upload/download HIS tools in editable formats and communicate with HIS teams, and 3) reporting on lessons learned afterwards to help improve best practices for future emergencies across organizations.

### Being better prepared for the next emergency

Even as the West African Ebola outbreak ended, the Zika virus outbreak in the Western hemisphere, cholera outbreaks in Yemen and parts of Sub-Saharan Africa, and the current Ebola outbreak in the Democratic Republic of Congo are reminders that epidemics will not wait while we design better systems. Yet an emergency setting is also the worst time to design a system.

Even minimal planning and time, though, can result in a more cohesive, comprehensive, and higher quality HIS if approached systematically. For example, we found that daily and weekly situation reporting requirements were very time-consuming, and initially entailed several hours of work each week. We were able reduce this work to minutes by developing written procedures, statistical software scripts for analyses, and templates for report writing. Using the list of inputs and outputs outlined in Table 2 could help HIS teams foresee possible future pitfalls and time sinks while still in the early stages of designing the HIS.

An emergency response organization would ideally have the core components of a high-quality HIS in place already, thereby only needing to adapt the system for a specific emergency. In Table 4, we provide a set of recommendations for organizations needing to design and implement an HIS during an emergency.

**Table 4:** Recommendations for designing and implementing the core components of a health information system during an emergency

Core HIS component	Recommendations
Overall	Use the inputs and outputs from Table 2 as a framework to design the HIS
	Create an overall plan of which system components will be needed, including priorities and thinking through contingencies
	Incorporate feasible evaluations (e.g. user questionnaires, comparison of records) into system planning from start if possible
	Create communication channels with other organizations to share HIS components throughout the emergency
Data collection tools	Investigate whether adaptable tools already exist through other organizations or in similar emergencies
	Pre-plan as many tools (e.g. forms, databases) as possible, and treat them as a unit
	Include error reduction techniques from the beginning (e.g. check digits)
Communication of data	Identify range of approaches that will allow data communication with speed, accuracy, and confidentiality
	Test different methods early and make sure they are working for all relevant parties
	Think unconventionally (e.g. plasticized paper) if needed
Coordination amongst relevant parties	Maintain communication between HIS and other relevant departments throughout the emergency
	Try to hire staff in leadership roles who can stay involved for a long period to minimize turnover
	Pre-plan handover strategies, including overlap timing between outgoing/incoming staff and written handover notes
Staff training	Create tools in advance that are easy-to-use and easily updated (e.g. simple PowerPoint slides) for training sessions
	Allow time for staff to become familiar with the data collection tools
	Ensure that training includes “do”s and “don’t”s for high-quality data collection based on tools in use
	Develop a plan for training, including training schedules, frequency of refresher trainings, and easy accessibility to HIS staff outside of regular training
	Make easy-to-access tools (e.g. laminated, annotated forms in the clinicians station) available in addition to training sessions
	Ensure training is done in as many languages as necessary for staff to be fully trained
Data management	Make and use databases for data digitization as early as possible
	Perform digitization in as close to real-time as possible
	Hire staff according to planned double entry during digitization if possible
	Ensure that all patient data are securely stored, and develop a plan in advance for what will happen to the patient records after the emergency ends
Data analysis/ reporting	Develop templates and analysis scripts for reports to internal and external actors
	Ensure that one master database is used for analyses

Evaluations, while often sidelined during emergencies, are important because they help move the field forward by providing a measure of what worked and what failed. The best likelihood of performing an evaluation is to plan for it during the system design phase. We planned our first evaluation questionnaire early and were thus able to implement it even months later. But planning for a Krio translation would have increased participation amongst non-English speaking local staff. Our vaguer plans for a follow-up questionnaire fell through because other urgent priorities meant we no longer had time later to both draft and implement it. Simple and fast evaluation strategies exist, and situations may arise that yield unintended opportunities. For example, we were able to 1) conduct a staff questionnaire (i.e. simple and fast) and 2) compare data that were collected using different methods as our protocols and systems changed (i.e. unintended opportunity).

Finally, this outbreak highlighted the need for appropriate standardized health record forms that can be used across organizations in an emergency, mitigating divergence between organizations. Divergence occurred during the West African Ebola outbreak partly because the available standardized forms were not designed for ETC red zones. Having any pre-existing form was helpful, though, and allowed organizations that adapted these tools to still collect relatively similar information. But minor differences in question wording can make data incomparable. Efforts to retrospectively combine data from different ETCs thus now have the more difficult task of trying to amalgamate less comparable data while maintaining data quality for research.

Standardized forms need a set of essential core questions, with flexible templates that permit the addition of organization-specific questions. The CDC’s case investigation form is an example of the benefits a standardized tool can provide [7], although even this form had to be simplified during the outbreak [26]. Ideally, future standardized data collection forms (and their associated training) would be developed based on lessons learned during this and other emergencies, feedback from organizations, and user testing. These tools must be openly available, and hosted by well-established organizations such as the CDC or WHO.

## Conclusions

Health information systems are often designed quickly and in an ad-hoc fashion during emergencies. Yet, thoughtful design and implementation is possible and can lead to more efficient and higher quality data collection for patient care, reporting, and research. The limitations imposed by onerous infection control for Ebola forced us to design health information systems that were focused on ease of use and efficiency. Many of the results and lessons learned from our experience are generalizable to non-Ebola emergencies. With systematic planning and design processes, alongside improvements during non-emergency times, high quality data collection in a low-resource health emergency can become a norm.

## Additional files

Additional file 1: Supplementary details for the main manuscript. (PDF 594 kb)

Additional file 2: Forms of the paper-based health information system used at the Kerry Town Ebola treatment center. (PDF 5350 kb)

## Abbreviations

EHR: Electronic health records
ETC: Ebola treatment center
HIS: Health information system
IT: Information technology
IV: Intravenous
MSF: Médecins Sans Frontières
PHR: Paper-based health records
PPE: Personal protective equipment
RFID: Radio-frequency identification
SCI: Save the Children International
UPS: Uninterruptable power supply
VHF: Viral hemorrhagic fever
WLAN: Wireless local area network
