# Supplementary material for: Improving health information systems during an emergency: lessons and recommendations from an Ebola treatment centre in Sierra Leone
Source: BMC Med Inform Decis Mak. 2019 May 27;19:100. doi: 10.1186/s12911-019-0817-9 (PMC6537453; doi:10.1186/s12911-019-0817-9)
Supplement: Supplementary file 1 — Supplementary details for the main manuscript. (PDF 594 kb) [file 12911_2019_817_MOESM1_ESM.pdf]

Improving health information systems during an emergency: lessons and recommendations from an Ebola Treatment Centre in Sierra Leone

Shefali Oza*, Kevin Wing, Alieu A. Sesay, Sabah Boufkhed, Catherine Houlihan, Lahai Vandi, Sahr Charles Sebba, Catherine McGowan, Rachael Cummings, Francesco Checchi

* corresponding author

Additional file 1: Supplementary details for the main manuscript

This document contains supplementary details, as referenced in the main manuscript, about the paper and electronic health information systems at Save the Children International's Kerry Town Ebola treatment center (ETC) in Sierra Leone from 2014-2015.

Table of contents

A1: Overview of the full Kerry Town ETC…………………………………………………………page 2
A2: Building a PHR and EHR in parallel……………………………………………………...………..page 3
A3: Staff questionnaire……………………………………………………………………………………..page 4
A4: EHR platform search…………………………………………………………………………………page 6
A5: Additional clinical workflow details…………………………………………………………...page 6
A6: Summary of paper-based health information system forms………………………...page 8
A7: Using check digits for patient ID numbers…………………………………………………..page 9
A8: Examples of revisions to HIS based on trial/error and user feedback……………..page 9
A9: Additional system usage results………………………………………………...………………page 11
A10: References………………………………………………………………………………………………...page 11

A1: Overview of the full Kerry Town ETC

We annotated a photograph of an aerial view of Save the Children's Kerry Town Ebola Treatment Center in Sierra Leone (Figure A1). This figure shows the locations of various ETC buildings, as well as the red and green zones. More generally, it shows the massive scale of this ETC.

Figure A1. The Kerry Town Ebola Treatment Centre in Sierra Leone (2014-2015) with annotations.

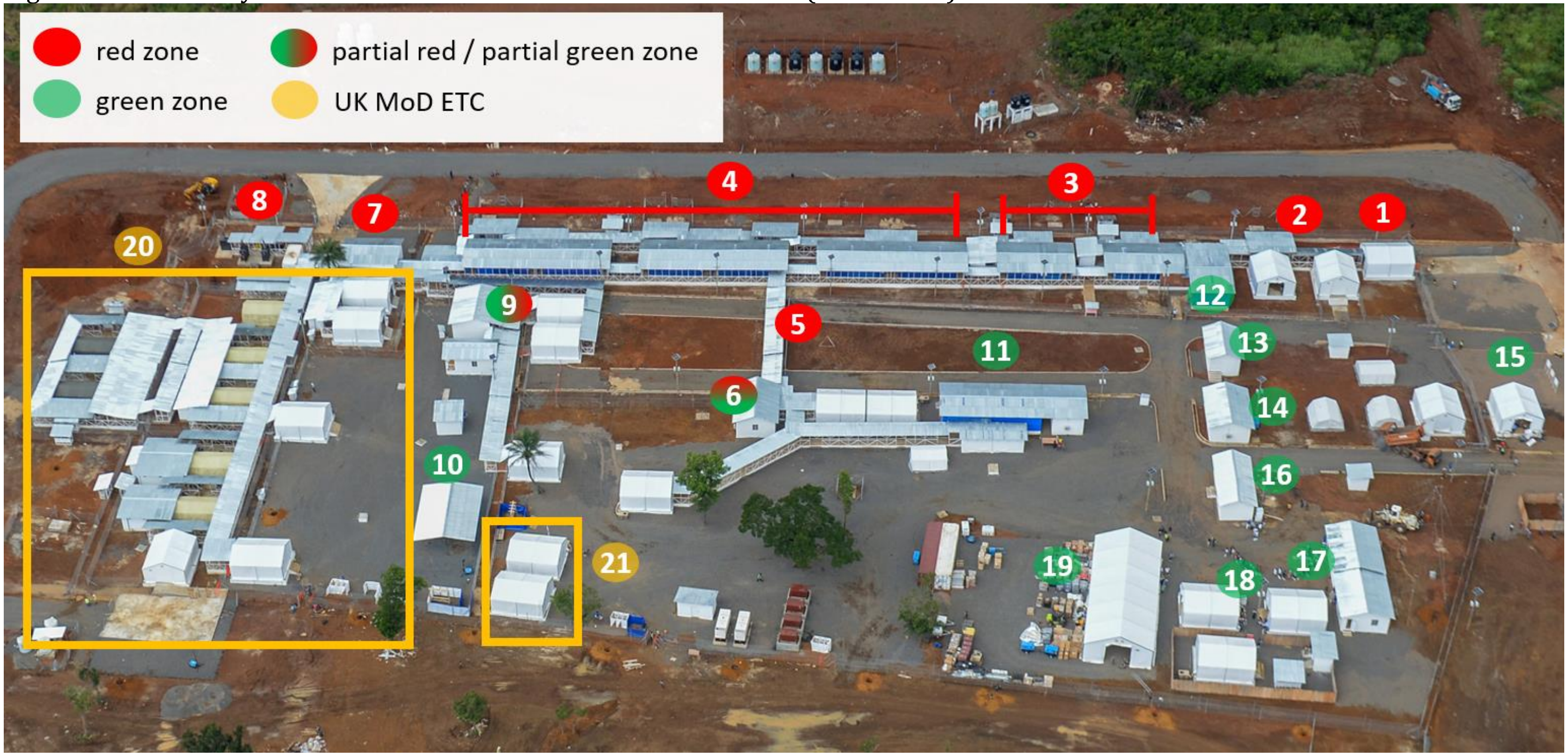

1 – patient entrance; 2 – triage; 3 – suspect wards; 4 – confirmed wards; 5 – walkway to Public Health England (PHE) laboratory; 6 – PHE laboratory; 7 – morgue; 8 – incinerators; 9 – decontamination area; 10 – laundry; 11 – offices (HIS, patient care, etc); 12 – clinicians' station/dressing room; 13 – doctors' room; 14 – pharmacy; 15 – training tent; 16 – operations (management) office; 17 – canteen/kitchen; 18 – clinician resting quarters; 19 – on-site warehouse; 20 and 21 – separate United Kingdom Ministry of Defense ETC for health workers

Photo credit and copyright information: This photo, taken by the UK Ministry of Defense, is available for public use under the Open Government License (http://www.nationalarchives.gov.uk/doc/open-government-licence/version/3/). We obtained this photo from the following URL: http://www.defenceimagery.mod.uk/fotoweb/archives/5042-Downloadable%20Stock%20Images/Archive/Royal%20Navy/45158/45158320.jpg

A2: Building PHR and EHR systems in parallel
A salient question is whether it was worth building an electronic health record (HER) system *during* the outbreak (particularly relevant as our ETC closed shortly after system implementation). With hindsight, we believe that it was worth building the EHR in our situation for several reasons. First, the communication challenges with paper-based data systems at ETCs were so severe that an EHR had unusually high potential for improving data communication, accuracy of patient records, and therefore clinical care in the ETC. Second, during the software development period, the epidemic was expected to last longer than it ultimately did [1-3]. Based on staff feedback and evaluation, we believe that use of the EHR system would have resulted in improvements in the quantity and quality of patient data if our site had stayed open longer. Additionally, our EHR could also have been adapted for use at other sites. Third, by using the existing OpenMRS platform, we were able to expedite development. Despite this, although 2.5 months is rapid for software development, it is slow for an emergency. Fourth, the cost was lower than similar software development; the estimated project cost of 260,000 USD, but the majority of this was donated as staff time by ThoughtWorks and others [4]. In total, SCI paid approximately 50,000 USD towards the EHR. This was thus a cost-effective choice for a software system, particularly for one that was built to be adaptable for future needs. Finally, while the resource and time costs were initially lower for the paper-based health record (PHR) system, they were lower for the EHR system in the later stages. For example, retrospective analysis and evaluation were much simpler with the EHR data, while the PHR system required many weeks of data entry and quality checks. This is not a trivial difference; such time savings and user-friendly data make monitoring the quality of data being collected *during* the emergency itself easier, which can improve both patient care and data quality. Similarly, although setting up the PHR database was relatively simple, it had limited patient information compared to the EHR because of 1) having to transcribe sometimes illegible handwritten patient records and 2) the amount of information in the paper records was too vast to enter by a small data entry team. For example, in the PHR database we included medications that were given to a patient but not the detailed information around dose, time of delivery, and whether the patient took all of the medication. This is all captured and transmitted instantaneously by the EHR system. Such nuanced details have the greatest potential for improving daily operations, especially when monitoring can be done automatically.

When contemplating both PHR and EHR systems as we did, careful considerations need to be made about parallel systems. Parallel systems will occur in two main scenarios: 1) during the transition from a PHR to EHR system (especially one that uses the phased development approach) and 2) if the EHR system is not a stand-alone system. Aside from the potential inefficiencies created by working with two different systems at the same time, there is a risk that introducing an EHR system may lead to confusion about the use of the existing (PHR) system. Thus, it is vital for the HIS team to work with clinicians to map out exactly how the transition between systems will work, and what if any information may still require paper documentation and viewing. Such transitions may also be important opportunities for evaluation. For example, having to keep parallel paper records for a few weeks while implementing the drug-ordering module of our EHR meant that we were able to perform an evaluation by comparing those records [4].

More generally, this work serves as a demonstration that an EHR can be built and implemented during an emergency in a low-resource setting. But as EHRs and the hardware required for them become more common throughout the world, we may soon be grappling with a different set of questions regarding such data collection. Thus, it is prudent to think carefully now about how best EHRs can be designed and used for complex low-resource emergency settings. The best-case scenario is a flexible EHR platform that can be adapted for a specific emergency within days or a few weeks, alongside inexpensive robust hardware that works well in low-power situations.

A3: Staff questionnaire
Here, we have included the pre-EHR questionnaire we developed to obtain clinician feedback on the Kerry Town HIS. For the purposes of inclusion in this appendix, we have removed the additional spacing we had provided for responses and have decreased the font size. The actual questionnaire was 4 pages due to the empty spaces for responses.

Date: ______________________

Kerry Town health information system usability survey

In this survey, we would like to ask you some questions about your experience with using the patient medical records at the Kerry Town ETC. The aim of this is to compare experiences with the existing paper based system to future experiences of an electronic system. We are particularly interested in your experience with the system as a whole instead of feedback regarding a specific form.

Basic information

1. What is your name (optional):
2. What is your job title:
3. How long have you been working at the Kerry Town ETC? ____________ ☐ months OR ☐ weeks
4. List your professional training:
5. Have you worked at an Ebola Treatment Centre prior to this job: ☐ yes ☐ no
   If yes, for how long:
6. Is English your first/primary language? ☐ yes ☐ no

General usability of medical records at Kerry Town ETC

1. Have you filled out any patient medical records at the Kerry Town ETC? ☐ yes ☐ no
2. Have you reviewed any patient medical records at the Kerry Town ETC? ☐ yes ☐ no
3. Please list 3 things you think are working with the current system:
4. Please list 3 things you think are not working with the current system:
5. In your experience, who primarily inputs information into the system?:

Please answer the following questions on a scale of **1 (poor) to 5 (excellent)** by circling the number of your choice, and provide a brief explanation for your choice.

6. Ease of recording patient information in the green zone: 1 2 3 4 5

   Please explain:

7. Ease of recording patient information in the red zone: 1 2 3 4 5

   Please explain:

8. Ability to review how a patient is doing: 1 2 3 4 5

   Please explain:

9. Ability to order drugs or IV fluids: 1 2 3 4 5

   Please explain:

10. Ability to track drugs that have been ordered: 1 2 3 4 5

Please explain:

11. Ability to track drugs that have been administered: 1 2 3 4 5

Please explain:

12. Ability to track fluid balance: 1 2 3 4 5

Please explain:

13. Ability to track vital signs: 1 2 3 4 5

Please explain:

14. Ability to track symptoms: 1 2 3 4 5

Please explain:

15. Ability to add any additional notes or documentation other than the above: 1 2 3 4 5

a. Please explain:

b. What types of additional notes do you add?

16. Please list additional comments/thoughts about the paper record system at the Kerry Town ETC:

Future electronic medical record system

We are looking to rollout an electronic/automated medical record system at the Kerry Town ETC. You may have already received some initial training on the first phase of this system. Our goal is to have a system that will include patient registration, baseline information, vital signs, symptoms, drug and IV fluid ordering, drug and IV fluid administration monitoring, and patient discharge.

Here we would like to ask you broad questions about a general electronic system and not the specific system you may have already seen.

1. Do you agree or disagree that an electronic patient record system could improve the patient record system at the ETC? (please circle your choice):

Strongly disagree Disagree Neutral Agree Strongly agree

Please explain:

2. Have you used an electronic medical record system or other health informatics system in the past? ☐ yes ☐ no

3. Please list 3 things you would like to see in an electronic system for Ebola:

4. Please list 3 advantages of an electronic instead of paper system in your opinion:

5. Please list 3 disadvantages of an electronic instead of paper system in your opinion:

6. Please list additional comments/thoughts about an electronic medical record system for the Kerry Town ETC:

Thank you for completing this survey! We are grateful for your help.

A4: EHR platform search

We searched (online and using word-of-mouth) for EHR platforms suitable for low-resource settings that could be adapted to our needs. In particular, we were seeking a platform that could 1) work in a low-resource setting, 2) be rapidly adapted as needed; 3) support complex EHR processes like drug ordering and IV fluid monitoring (which were deemed among our top priorities for an EHR) [4], and 4) ideally (but not required) be open-source. Our main priority for the EHR was to solve the communication issues we were having with transferring patient data from the red to green zone. After looking at the available options, we found that no pre-existing platforms directly met our requirements. We contacted several people, organizations, and companies which we believed may have software we could adapt to our needs. Of the software platforms we reviewed, some were cloud-based. Given that our satellite dish for Internet was unreliable and slow, cloud-based options were not feasible for us. Some platforms lacked the flexibility we needed to design interfaces for red zone use or lacked the complexity we needed for modules like drug ordering. While simpler software was being used for other tasks during the outbreak, such software was not relevant for our demands in the red zone. To our knowledge, this is still the case even in the current Ebola outbreak in the Democratic Republic of the Congo. Software such as Open Data Kit (ODK) is useful for various data collection needs, but cannot substitute for more complex EHR modules. Ultimately, the closest fit appeared to be adapting the already established open-source OpenMRS software [4, 6]. This module-based platform was rapidly adaptable and had the complexity required for some of our EHR needs. Additionally, by choosing an open-source platform and making our development open as well, we aimed to build tools that could be broadly shared across ETCs without the intellectual property barriers posed with proprietary software. We have published details about this software development elsewhere [4].

A5: Additional clinical workflow details

Here, we have included additional details of the clinical workflow at the site. The description below is an example of the initial clinical workflow. Some of these details changed over the course of the ETC operations, largely due to changes in clinical leadership and/or protocols.

- The daily medical shifts consisted of clinical teams including doctors, nurses, and community health officers. Individuals on each team were partnered because of infection control regulations.
- Patient summary data was on large boards in the clinicians' station next to the entrance to the red zone. Boards were organized by ward number and bed number. Board information included key relevant details: patient name (later changed to only ID number), date of admission, date of diagnosis, stage of illness or symptoms, malaria result and date, other diagnosis (e.g. concurrent malaria, pregnancy, acute renal failure etc), last abnormal bloods (and data), current drugs, current fluids (IV / oral), presence of IV access (yes/no) and planned discharge.
- Each shift duration included a 30-minute overlap between teams. During this handover, the team would discuss each patient individually highlighting things which were needed on that shift. Handovers were led by clinician in charge of each shift or the clinical lead for the ETC.
- Each patient would be seen at least once a day on a general ward round, usually during the morning shift.
- In the red zone, each patient had a file which contained clinical notes, observations, fluid charts and prescription / drug administration charts. These were stored at one end of each ward, organized by bed number. These records were later scanned in the red zone using a WiFi-enabled scanner and then incinerated.
- Information from inside the ward would be communicated to the clinical office in ways detailed elsewhere in the paper (predominantly radio and collective memory).

- Each patient had a paper health record in the clinicians' station which included a prescription sheet, fluid charts, daily inpatient forms, and other relevant documents (e.g. intake form). After exiting the red zone and removing PPE, team members would come back to the clinicians' station and update patient notes with what information they could remember or review information entered by the person who had received radio information. They would verbally hand over tasks to the next team member entering the red zone and update the master board.
- Blood results would be printed out by the laboratory team and deposited in a box in the clinicians' station. This would be reviewed and signed by a medical team member and filed in the patient's folder in the clinician's station. Important or urgent results would be written on the master board.
- Pharmacists would come to the clinicians' station to review the prescription charts in the patient files, and would keep duplicate patient pharmacy records in the pharmacy.
- Blood request forms were written before entering the red zone and deposited in the laboratory reception in the red zone with the request form.

The EHR workflow was designed to mimic the PHR workflow in terms of data being collected. The tablet-based EHR modules mimicked the data collected using the PHR in the red zone (e.g. drug ordering, IV fluid monitoring, vitals/signs, symptoms). The desktop-based EHR modules mimicked the green zone PHR data collection (e.g. registration, laboratory results, bed location, additional patient notes). Similar to the PHR red zone records, the tablets used for EHR data collection were also housed in the patient wards.

A6: Summary of paper-based health information system forms

Table A1: Data collection forms for the Kerry Town ETC paper-based health information system

Paper form	Description	Rollout date	Location[1]
**Baseline information**			
**Triage**	Symptom and exposure information to determine if patient meets case definition for admission to ETC as suspect EVD case	19/01/2015[2]	Triage
**Case investigation**[3]	Standardized form with demographic, symptom, and epidemiologic risk factor information	19/01/2015	Triage or previous holding center
**Ward assessment**	Baseline Kerry Town form for new patients (selected information on demographics, symptoms, vitals/signs, medical history)	05/11/2014	Ward, Clinician station
**Clinical forms**			
**Inpatient**	Form used at least daily to record observations, signs, symptoms, and clinician notes)	05/11/2014	Ward, Clinician station
**Drug charts**	Prescription and monitoring chart with drug name, date, route, dose, frequency, prescriber details	05/11/2014	Ward, Clinician station
**Fluid infusion chart**	Chart with infusion fluid, date, volume, rate, route, timing, checks, clinician details	28/11/2014	Ward, Clinician station
**Lab request**	Form to request Ebola PCR and rapid malaria test from the Public Health England (PHE) laboratory or biochemistry tests from the UK Ministry of Defense (MoD) Laboratory	05/11/2014	Ward
**Lab results**	Printouts from the PHE and MoD laboratories with patient test results	05/11/2014	Clinician station
**Discharge form**	Information related to death, discharge, or transfer from ETC	05/11/2014	Ward, Clinician station
**Administrative forms**			
**Nutrition summary**	From provided to kitchen detailing type of food patient was able to eat (e.g. solid, semi-solid, liquid)	19/11/2014	Clinician station
**Exit form**	Details of discharge (e.g. mode of transport), follow-up (e.g. contact information), and checklist for discharge packet	05/11/2014	Discharge tent
**Patient evaluation**	Completed by patient upon discharge regarding opinion of ETC stay (e.g. medical care, food, support, staff support)	01/01/2015	Discharge tent
**Discharge certificate**	Certificate provided to discharged survivor stating that s/he is Ebola-free	09/12/2014	Patient care office
**Death certificate**	Certificate provided to family of dead Ebola-positive patient	09/12/2014	Patient care office
**Additional patient information**			
**Clinicians log book**	Notebook detailing essential patient information by ward (e.g. symptoms, medications, notes)	05/11/2014	Clinician station
**Cuban log book**	Notebook detailing essential patient information by ward for Cuban clinicians (in Spanish)	05/11/2014	Clinician station
**Holding centre forms**	Forms from previous holding centers, typically containing basic demographic and symptom data	N/A[4]	Clinician station

[1] This refers to the location where data was collected for each form. Some data were collected in the red zone when possible, but also in the green zone at the Clinicians station through radio transmission from the red zone or collective memory by clinicians once their ward round was completed.

[2] This is the date that the Kerry Town ETC officially began accepting suspected EVD patients.

[3] This was a standardized Centers for Disease Control and Prevention (CDC) form provided to us. We completed it for our suspect patients once the suspect wards officially opened at the ETC. For confirmed EVD patients arriving from previous holding centers where the form was completed (including before our suspect ward was opened), we retained a duplicate version of the form.

[4] N/A = Not applicable. We retained forms provided by previous holding centers for confirmed EVD patients transferred to our site.

A7: Using check digits for patient ID numbers

We used a check digit as the final number for each patient ID as a basic way to prevent ID errors. We ran a check digit algorithm in advance of the ETC opening and created thousands of potential ID numbers. As an illustration of how check digits work, the first five patient IDs using the algorithm could have been KT-3-00008, KT-3-00017, KT-3-00024, KT-3-00032, and KT-3-00044. The first 4 digits of each ID number are sequential, the last digit is random. This meant that if a user miswrote a digit in the ID number, the likelihood of this being a real ID number for another patient is very low.

After running the algorithm, we printed out sheets of thousands of check digit ID numbers selected by the algorithm with the Kerry Town ID number format. We then pre-wrote patient ID bracelets with these IDs, added the ID bracelets to a box, and picked one at random for each newly registered patient. This further decreased the likelihood of ordered patient ID numbers. This system relied on HIS involvement to assist the clinicians in preparing the ID bracelets. Even if the random selection of patient ID bracelets was not done, the check digit still meant ID mix ups that were unresolvable were unlikely.

See https://wiki.openmrs.org/display/docs/Check+Digit+Algorithm for a check digit algorithm.

A8: Examples of revisions to HIS based on trial/error and user feedback

Here, we provide some additional examples of revisions we made to the HIS.

Red to green zone communication

1. Wi-Fi scanner. We initially planned for a Wi-Fi scanner to be used in the red zone to transfer red zone patient records to the green zone over our ETCs Wi-Fi network. For this and our planned EHR, we made certain that the red zone had Wi-Fi capabilities before the ETC opened and made plans for how and when patient records would be scanned and by whom. Then, due to unexpected supply chain delays, we did not receive the Wi-Fi scanner for over two months after the site opened. This delay forced us to change our red to green zone communication plans, relying on radio and "collective memory" (as described in the main paper) instead. The new medical director, who arrived around the same time as we received the scanner, was not interested in introducing the scanner into the red zone. This likely would not have occurred if the scanner had already been part of the workflow.

2. Photographs of summary data. We investigated several methods of red to green zone communication. Several methods used by other organizations, including shouting information to the green zone, were not feasible for us because of the enormous distances between some of the red zone wards and the nearest green zone point. But we still installed hallway white boards in hopes of taking photographs of patient information, which proved unsuccessful even with a good camera lens. We looked into ordering plasticized paper but the supply chain issues we were facing made this an unreliable option.

   Out of desperation, we attempted the following time-consuming communication method for 3 days. First, a clinician would summarize patient information for 10 patients (i.e. one ward) on a piece of paper containing boxes for [[[xxxxx]]] based on information in the green zone clinicians' station patient charts. Then, the clinician would take this sheet into the red zone, and would record new information (or correct errors) in a different colored pen once inside the red zone. A clinician would then bring this sheet out to the decontamination area, where an HIS team member would be waiting. The clinician, still on the red zone side of the decontamination

area, would hold up the sheet of paper, which the HIS team member would photograph (Figure A2). The HIS team member would then upload these photographs and print them to bring to the clinicians station. This method was inefficient and time-consuming, but we attempted it out of desperation for a better communication method. We decided after three days to not pursue this because of its impracticality.

Figure A2. Attempted (and ultimately rejected) method to communicate summary of individual patient information from red to green zone.

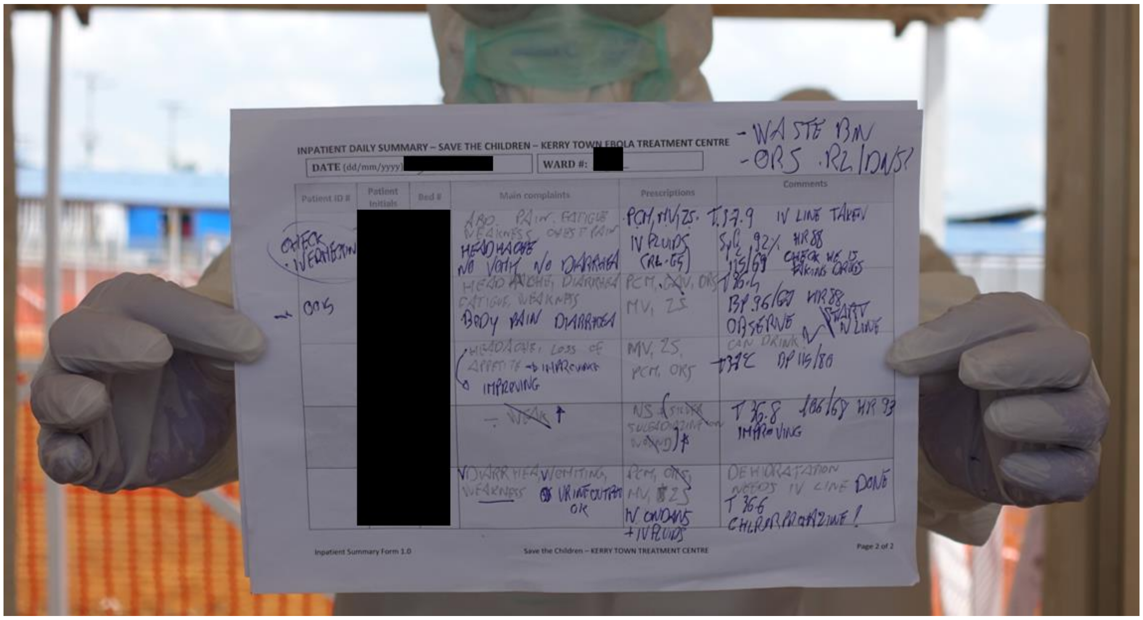

Photo by Shefali Oza

Changes to the paper-based health information system

1. To build the backbone of our paper-based health records, we adapted the ISARIC case record form [7] based on advice from the medical director. The ISARIC form in its original form was not appropriate for a red zone environment (e.g. very small font sizes, too many complex questions). We sought advice from the medical lead and other medical advisors on which questions to keep or delete to have an essential/minimal dataset. But the result from these discussions was still a form that was overly long and complex, including questions that were deemed essential by the advisors but not practical for the setting (e.g. capillary refill time, O_2 saturation, hepatomegaly/splenomegaly in centimeters). We had to iterate over the form two more times to remove additional questions based on user feedback and increase our already large font sizes further after testing with goggles.

2. The clinical workflow when the site started included drug ordering in the red zone. The difficulty in communicating red zone drug orders to the green zone accurately was a key reason for our building of an EHR in parallel to the PHR. The workflow involved radioing in drug orders to the clinicians' station, where it would be documented in a patient chart and subsequently communicated to the pharmacy (which created a duplicate patient drug chart). Over time, this work flow shifted to one where drug orders were planned ahead of time in the green zone. While this was non-ideal compared to bedside ordering, given the challenges of red to green zone communication, this was considered a better option. This change in workflow affected which data were collected where.

3. Sometimes changes to forms were proposed months after they had been put into circulation. We were frequently making decisions like these on whether or not to move forward with a revision – balancing the push for changes (especially by newly arriving international staff) with the stability of a system on which users (especially long-term staff) were trained. Even when we agreed with a proposed change, we tended to reject the change in favor of system stability if it was not clear how users would be adequately trained. The exception would have been if a change was essential for the system, but most of those had been discovered within the first several weeks of having the site open.

A9: Additional system usage results

See "System usage" in the results section of the main paper for further context.

Reasons for missing data/files

For the five (of 456) patients with missing additional patient files (i.e. beyond the basic demographic, Ebola status, outcome information), the files were missing because of incomplete form completion or the files being lost during storage. Four of these patients died or were discharged less than 24 hours after admission and one was admitted on the day the ETC opened (i.e. when the HIS system was new). For the 14 of 456 patients with missing medication forms, eight died or were discharged less than 24 hours after admission and two were admitted on the first day of the ETC opening. Twenty-seven patients (5.9%) had missing baseline symptom information, likely due to a combination of admission on the first day, incomplete forms, and lost files during storage.

Quantity of patient data recorded and/or stored

The median number of pages (with interquartile range) of health records for Ebola-positive patients was 18 (11-28) for those who died and 24 (15-39) for those who recovered. This difference corresponds to a similar difference in the median length of stay at the ETC by outcome: 3 (2-5) days for Ebola-positive patients who died and 9 (6-14) days for those who recovered.

A10. References

[1] Chan M. Ebola virus disease in West Africa—no early end to the outbreak. *New England Journal of Medicine.* 2014;371(13):1183-1185.

[2] Meltzer MI, Atkins CY, Santibanez S, et al. Estimating the future number of cases in the Ebola epidemic—Liberia and Sierra Leone, 2014–2015. *MMWR Surveill Summ.* 2014;63(Suppl 3):1-14.

[3] Aylward B, Barboza P, Bawo L, et al. Ebola virus disease in West Africa--the first 9 months of the epidemic and forward projections. *N Engl J Med.* 2014;371(16):1481-1495.

[4] Oza S, Jazayeri D, Teich JM, et al. Development and Deployment of the OpenMRS-Ebola Electronic Health Record System for an Ebola Treatment Center in Sierra Leone. *J Med Internet Res.* 2017;19(8):e294.

[5] Jobanputra K, Greig J, Shankar G, et al. Electronic medical records in humanitarian emergencies– the development of an Ebola clinical information and patient management system. *F1000Research.* 2016;5.

[6] OpenMRS. OpenMRS website. URL: http://openmrs.org. Archived at http://www.webcitation.org/6pKc70Npm.

[7] ISARIC, WHO. ISARIC WHO CCP VHF Case Record Form v7.0 10 August 2014. 2014; https://isaric.tghn.org/site_media/media/medialibrary/2016/06/ISARIC_WHO_CCP_VHF_Case_Record_Form_v7.0_10AUG14.docx. Accessed 2017/08/28.
