# Supplementary material for: Improving health information systems during an emergency: lessons and recommendations from an Ebola treatment centre in Sierra Leone
Source: BMC Med Inform Decis Mak. 2019 May 27;19:100. doi: 10.1186/s12911-019-0817-9 (PMC6537453; doi:10.1186/s12911-019-0817-9)
Supplement: Supplementary file 2 — Forms of the paper-based health information system used at the Kerry Town Ebola treatment center. (PDF 5350 kb) [file 12911_2019_817_MOESM2_ESM.pdf]

Improving health information systems during an emergency: lessons and recommendations from an Ebola Treatment Centre in Sierra Leone

Shefali Oza*, Kevin Wing, Alieu A. Sesay, Sabah Boufkhed, Catherine Houlihan, Lahai Vandi, Sahr Charles Sebba, Catherine McGowan, Rachael Cummings, Francesco Checchi

* corresponding author

Additional file 2: Forms for the paper-based health record system used at the Kerry Town Ebola treatment center

This document contains the forms used at the Save the Children International's Kerry Town Ebola treatment center in Sierra Leone from 2014-2015. A description of the forms and information that comprised the paper-based Kerry Town health information system is provided in section A6 of "Additional file 1". Together, these forms encompassed demographic, epidemiological, clinical, and treatment information for individual patients, as well as information for management such as death certificates.

The majority of forms listed below were developed or adapted by us for use in the infectious wards (i.e. red zone) of the Kerry Town Ebola treatment center (ETC). The case investigation form was a standardized CDC/WHO form that we used as is. Some of these forms were revised over time; the documents here are the last versions used at the ETC.

The forms are listed below. Before each form is a descriptive page about the form.

1. Triage form……………………………………page 2
2. Case investigation form…………………..page 4
3. Ward assessment form…………………..page 7
4. Inpatient form……………………………….page 11
5. Drug charts……………………………………page 14
6. Fluid infusion chart………………………..page 18
7. Lab request…………………………………...page 20
8. Nutrition summary form………………..page 22
9. Discharge forms…………………………….page 24
10. Exit form………………………………………page 28
11. Patient evaluation form…………………page 30
12. Discharge certificate……………………..page 32
13. Death certificate……………………………page 34

Triage form

The following form was used by staff to determine if the patient met the case definition for Ebola. If s/he did meet the case definition, then the patient was admitted to a suspect ward and tested for Ebola. If s/he did not meet the case definition, then the patient was not admitted to the Kerry Town Ebola treatment center.

Triage Form

Date: ____ /____ / 2015

Name of patient: ________________________________ Sex: Male ☐ Female ☐ Age:______ Address: ____________________

____

Clinical Status at Visit

- **Well** → Not Suspect Case (Tick ☐)
- **Not Well** → both checklists below

Does the patient have fever greater than 38° ☐

Or a history of self-reported fever ☐

AND 3 or more of the following:

Headache	☐	Difficulty breathing	☐
Loss of appetite	☐	Nausea/Vomiting	☐
Fatigue	☐	Abdominal Pain	☐
Joint/Muscle pain	☐	Difficulty Swallowing	☐
Diarrhoea	☐	Hiccups	☐
Unusual bleeding	☐		

→ Suspected Ebola case: Admit! (Tick ☐)

In the last 3 weeks has the patient done any of these:

Cared for or been cared for by a sick person	☐
Wash the clothes of a person who was sick or has died	☐
Slept with someone who was sick or has died	☐
Touched the body of someone who was sick or has died	☐
Washed the body of someone who has died	☐
Attended the funeral of someone who has had Ebola	☐
Touched a sick or dead monkey or bat	☐
Breastfed from a sick person	☐

→ Suspected Ebola case: Admit! (Tick ☐)

No to both: Not Ebola Case (Tick ☐)

Case investigation form

The following form was a standardized form from the CDC/WHO that was either completed by us (for new suspect patients who did not yet have an Ebola test) or by a previous holding center (for confirmed patients who tested positive elsewhere and were transferred to our center for treatment). The information from this form was then sent to the government and

EBOLA CASE INVESTIGATION FORM - Sierra Leone

Outbreak Case ID:

Date of Case Report: ______/______/______(DD,MM,YYYY)

☐ Patient is a followed contact*: **Convert to CASE in VHF***
**Complete at end of interview:**☐ suspect ☐ probable ☐ unk

Patient's Last Name: ________________________ **First Name:** _____________________________

Age: ______ Unit: ☐Years ☐Months **Gender:** ☐ Male ☐ Female

Patient Status at Time of This Report: ☐Alive ☐ Dead ***If dead***, Date of Death: ____/___/____ (DD,MM,YYYY)

Permanent Residence:

Head of Household: _________________________ Village/Town: ___________________________

District: ___________________ Chiefdom: __________________ Mobile phone #: __________________

Patient's Occupation:

☐ Healthcare worker (includes anyone involved with the patient: nurse, ambulance driver, hospital cleaner, etc.)

Position: _________________ Healthcare facility: ________________

☐ Other; please specify occupation: ___________________________________

Location Where Patient Became Ill:

Village/Town: ____________________ District:_____________________ Chiefdom: ____________________

Date Patient First Became Sick: ______/_____/________ (DD,MM,YYYY)

Read each one aloud and mark an answer for every symptom occurred during this illness (not only right now):

Fever	☐ Yes	☐ No	☐ Unk	Headache	☐ Yes	☐ No	☐ Unk
Vomiting/nausea	☐ Yes	☐ No	☐ Unk	Difficulty breathing	☐ Yes	☐ No	☐ Unk
Diarrhea	☐ Yes	☐ No	☐ Unk	Difficulty swallowing	☐ Yes	☐ No	☐ Unk
Conjunctivitis (red eyes)	☐ Yes	☐ No	☐ Unk	Skin rash	☐ Yes	☐ No	☐ Unk
Intense fatigue/weakness	☐ Yes	☐ No	☐ Unk	Hiccups	☐ Yes	☐ No	☐ Unk
Anorexia/loss of appetite	☐ Yes	☐ No	☐ Unk	Unexplained bleeding	☐ Yes	☐ No	☐ Unk
Abdominal pain	☐ Yes	☐ No	☐ Unk	If yes, please specify:	___________________		
Muscle pain	☐ Yes	☐ No	☐ Unk	Other symptoms:	☐ Yes	☐ No	☐ Unk
Joint pain	☐ Yes	☐ No	☐ Unk	If yes, please specify:	___________________		

At the time of this report, is the patient hospitalized or being admitted to the hospital?☐ Yes ☐ No ☐ Unk

If yes, Date of Hospital Admission: _____/_____/______ (DD,MM,YYYY)

Hospital Name: ___________________________ District:_________________________________

Is the patient now, or will he/she soon be, in an Ebola treatment unit? ☐ Yes ☐ No ☐ Unk

If yes, date of admission (or future admission) to the ETU (isolation): _____/_____/______(DD,MM,YYYY)

Was the patient hospitalized or visit a clinic previously for this illness (this includes any type of care: pharmacist, traditional healer, etc.)? ☐Yes ☐No ☐Unk

If yes, Dates of Hospitalization: ________/_____/______(DD,MM,YYYY)

Hospital Name: ___________________________ District:_________________________________

IN THE PAST ONE (1) MONTH PRIOR TO SYMPTOM ONSET:

1. Did the patient have contact with a suspected or confirmed Ebola case in the one month before becoming ill?

☐ Yes ☐ No ☐ Unk

If yes, please complete one line of information for each sick source case:

Name of Source Case	Relation to Patient	Date of Last Contact (DD,MM,YYYY)	Village/Town	District	Was the person dead or alive ?
		___/___/___			☐ Alive<br>☐ Dead<br>Date of Death:<br>___/___/___<br>(DD,MM,YYYY)
		___/___/___			☐ Alive<br>☐ Dead<br>Date of Death:<br>___/___/___<br>(DD,MM,YYYY)

2. Did the patient attend a funeral in the one month before becoming ill? ☐ **Yes** ☐ **No** ☐ **Unk**

If yes, Name of deceased person	Relation to Patient	Date of Funeral (DD,MM,YYYY)	Village/Town	District	Did the patient participate? (carry or touch the body)?
		___/___/___			☐ Yes<br>☐ No

3. Did the patient travel outside their hometown or village/town before becoming ill? ☐Yes ☐No ☐ Unk

If yes, Village: ________________ Chiefdom: ____________________

District: ________________ Date(s): ______/______/______- ______/______/______ (DD,MM,YYYY)

Case Report Form Completed by:

Name: ______________ Phone: ______________ E-mail: ______________

Position: ______________ District: ______________ Health Facility: ______________

Information provided by:

☐ Patient ☐ Proxy *If proxy*, Name: ________________ Relation to patient: ________________

Patient Outcome Information:

Please fill out this section at the time of patient recovery and discharge from the hospital OR patient death.

Date Outcome Information Completed: ______/______/______(DD,MM,YYYY)

Final Status of the Patient: ☐ Alive/Recovered ☐ Dead

If the patient has recovered and been discharged from the hospital:

Hospital discharged from: ____________________ District: ____________________

Date of discharge from the hospital: /____/ (DD,MM,YYYY)

If the patient is dead:

Date of Death: ____/____/____(DD,MM,YYYY)

Place of Death: ☐ Community ☐ Hospital __________ District: ______________

Date of Funeral/Burial: ____/_____/______ (DD,MM,YYYY)

Funeral conducted by: ☐ Family/community ☐ Outbreak burial team

Place of Funeral/Burial: Village: __________ Chiefdom: __________ District: __________

Case ID: ________________

Ward assessment form

The following form was used by clinical staff to record baseline information for newly admitted patients (suspect and confirmed) to the Kerry Town Ebola treatment center.

SAVE THE CHILDREN – KERRY TOWN EBOLA TREATMENT CENTRE
WARD ASSESSMENT FORM (version 3.5)

Form completed by (write your name): ____

BASIC PATIENT INFORMATION

Patient name: Surname ______________________ First name ______________________

Address: District _______________ Chiefdom_____________ Town_______ ____________

Sex: ☐Male ☐Female **Other patient ID # (eg transfer facility):** ____________________

Estimated age: [ ] ☐YEARS ☐MONTHS (for children under 1 year)

ADDITIONAL PATIENT INFORMATION

Can patient eat: ☐ Nothing ☐ Liquid only ☐ Semi-solid food ☐ Solid food

***If Estimated Age is under 12 months (1 year)*:**

Currently breastfed? ☐YES ☐NO ☐Unknown

Next of kin: Name _____________________________ **Mobile #** ______________________

Address: District ______________________ Town/village ______________________

OBSERVATIONS

Disease stage: ☐ **1** (e.g. dry) ☐ **2** (e.g. diarrhoea/vomiting) ☐ **3** (e.g. shock, bleeding)

Current consciousness: ☐**A** ☐**V** ☐**P** ☐**U** **Confused/agitated:** ☐YES ☐NO

Clinically shocked? ☐YES ☐NO ☐UNKNOWN

Temperature: [___][___].[___]°C

Heart Rate: [___][___][___]beats /min **Respiratory Rate:** [___][___]breaths /min

Weight (kg) |___|___|.|___|

***If child under 5 years*, Mid Upper Arm Circumference (cm):** |___|___|.|___|

Systolic BP: [___][___][___]mmHg **Diastolic BP:** [___][___][___]mmHg

O$_2$ saturation: [___][___][___]% **On:** ☐Room air ☐Supplemental Oxygen ☐Unknown

DATE: ___ / ___ / 2015
DD / MM / YYYY

PATIENT ID #: KT–[ ] –[ ][ ][ ][ ][ ]
KT-2 = Triage; KT-3 = Confirmed; KT-4 = morgue

SYMPTOMS

Number of days since earliest onset of symptoms: ____ days

**Fever** ☐	**Hiccups** ☐
**Fatigue** ☐	**Convulsions** ☐
**Headache** ☐	**Seizures** ☐
**Joint or muscle pain/aches** ☐	
**Throat pain/Pain to swallow** ☐	***If BLEEDING,*** **specify site:**
**Unable to drink** ☐	**Nose/mouth** ☐
**Nausea** ☐	**Cough** ☐
**Vomiting** ☐	**Vomit** ☐
**Diarrhoea** ☐	**Urine** ☐
**Abdominal pain** ☐	**Stool** ☐
**Breathing difficulty** ☐	**Vaginal** (non-menstrual) ☐

Specify other symptoms:

OTHER MEDICAL HISTORY

Does the patient CURRENTLY have any known co-morbidities? tick all that apply

☐ Tuberculosis ☐ HIV ☐ Heart disease ☐ Diabetes ☐ Asthma

☐ Liver disease ☐ Renal disease ☐ Cancer

Other: ____

Does the patient have any known allergies? ☐ YES ☐ NO ☐ UNKNOWN

If YES, List____

If patient is FEMALE:

Date of last menstruation_____________ ☐ Unknown

Obstetric history: Gravida [__] **Parity** [__] **Abortion** [__] **Live** [__] **Dead** [__]

Has the patient ever had a Caesarean section? ☐ YES ☐ NO ☐ UNKNOWN

Is the patient currently breastfeeding? ☐ YES ☐ NO ☐ UNKNOWN

Is the patient: ☐ Pregnant ☐ Postpartum (birth in last 6 weeks) ☐ Neither ☐ Unknown

If PREGNANT: **Gestation age of fetus** (nearest week): ____ weeks ☐ Unknown

DATE: ___ / ___ / 2015
DD / MM / YYYY

PATIENT ID #: KT–☐ –☐☐☐☐☐
KT-2 = Triage; KT-3 = Confirmed; KT-4 = morgue

WARD ASSESSMENT FORM (version 3.5)

PRE-EXISTING MEDICATIONS

List all medications patient is taking/prescribed prior to admission (e.g. antibiotics, antivirals, antifungal, antimalarials, analgesic/antipyretics)

**Name of medication** *(prefer generic name)*	**Dose and frequency**
	☐unknown
	☐unknown
	☐unknown

SIGNS

**Bruising**	☐Yes	☐No	**Conjunctival injection**	☐Yes	☐No
**Pale/Anaemia**	☐Yes	☐No	**Hepatomegaly**	☐Yes	☐No
**Jaundice**	☐Yes	☐No	**Splenomegaly**	☐Yes	☐No
**Rash**	☐Yes	☐No			

Other (please specify)

ADDITIONAL COMMENTS

DATE: ___ / ___ / 2015
DD / MM / YYYY

PATIENT ID #: KT–☐ –☐☐☐☐☐
KT-2 = Triage; KT-3 = Confirmed; KT-4 = morgue

Inpatient form

The following form was used by clinical staff to record patient vital signs, observations, symptoms, and notes at least daily.

INPATIENT FORM (version 3.0)

***BEGIN NEW FORM ONCE THIS ONE IS COMPLETE**

OBSERVATIONS and SIGNS

Date: DD/MM		__ __/__ __	__ __/__ __	__ __/__ __	__ __/__ __
Time: 24 hr		____ : _____	____ : _____	____ : _____	____ : _____
# of days since admission					
Provider (your) name					
CURRENT Consciousness		A V P U	A V P U	A V P U	A V P U
Temperature °C					
Oxygen saturation (%)					
Respiratory rate breaths/minute					
Heart rate beats/minute					
Systolic BP mmHg					
Diastolic BP mmHg					
Pale/Anaemia		☐None ☐Some ☐Severe	☐None ☐Some ☐Severe	☐None ☐Some ☐Severe	☐None ☐Some ☐Severe
Hydration	Dehydration	☐None ☐Some ☐Severe	☐None ☐Some ☐Severe	☐None ☐Some ☐Severe	☐None ☐Some ☐Severe
	Vomiting	☐None ☐Some ☐Severe	☐None ☐Some ☐Severe	☐None ☐Some ☐Severe	☐None ☐Some ☐Severe
	Diarrhoea	☐None ☐Some ☐Severe	☐None ☐Some ☐Severe	☐None ☐Some ☐Severe	☐None ☐Some ☐Severe

SYMPTOMS (continued on next page)

Overall symptoms	☐Same ☐Better ☐Worse	☐Same ☐Better ☐Worse	☐Same ☐Better ☐Worse	☐Same ☐Better ☐Worse
Fatigue	☐None ☐Some ☐Severe	☐None ☐Some ☐Severe	☐None ☐Some ☐Severe	☐None ☐Some ☐Severe

Ward #: _____ Bed #: ______

PATIENT ID #: KT–☐ –☐☐☐☐☐
KT-2 = Triage; KT-3 = Confirmed; KT-4 = morgue

INPATIENT FORM (version 3.0)

Date: DD/MM	______ / ______	______ / ______	______ / ______	______ / ______
Symptoms continued	☐Fever<br>☐Headache<br>☐ Joint/muscle pain<br>☐ Throat pain/ pain to swallow<br>☐ Chest pain<br>☐ Short of breath<br>☐ Cough<br>☐ Hiccups<br>☐ Rash<br>☐ Abdominal pain<br>☐ Urine pain<br>☐ Bleeding<br>Other symptoms (list):<br><br>☐ NO SYMPTOMS	☐Fever<br>☐Headache<br>☐ Joint/muscle pain<br>☐ Throat pain/ pain to swallow<br>☐ Chest pain<br>☐ Short of breath<br>☐ Cough<br>☐ Hiccups<br>☐ Rash<br>☐ Abdominal pain<br>☐ Urine pain<br>☐ Bleeding<br>Other symptoms (list):<br><br>☐ NO SYMPTOMS	☐Fever<br>☐Headache<br>☐ Joint/muscle pain<br>☐ Throat pain/ pain to swallow<br>☐ Chest pain<br>☐ Short of breath<br>☐ Cough<br>☐ Hiccups<br>☐ Rash<br>☐ Abdominal pain<br>☐ Urine pain<br>☐ Bleeding<br>Other symptoms (list):<br><br>☐ NO SYMPTOMS	☐Fever<br>☐Headache<br>☐ Joint/muscle pain<br>☐ Throat pain/ pain to swallow<br>☐ Chest pain<br>☐ Short of breath<br>☐ Cough<br>☐ Hiccups<br>☐ Rash<br>☐ Abdominal pain<br>☐ Urine pain<br>☐ Bleeding<br>Other symptoms (list):<br><br>☐ NO SYMPTOMS

Comments				

Ward #: _____ Bed #: ______

PATIENT ID #: KT–☐ –☐☐☐☐☐
KT-2 = Triage; KT-3 = Confirmed; KT-4 = morgue

Drug charts

The following drug charts were used by clinicians and pharmacists to record and review medication prescriptions, as well as monitor medication administration dates/times. The first is a blank form for up to four medications. The second is an adult drug chart with three common medications (zinc sulphate, multivitamin tablets, and paracetamol) already filled in. The third is a similar pre-filled chart for pediatric patients, with zinc sulphate, multivitamin tables, paracetamol, and vitamin A.

Blank Drug Chart v3.6 Patient name: ______________________ Patient ID: KT– ☐– ☐☐☐☐☐

Complete **DRUG INFORMATION** (drug name, route, dose, frequency, your name, signature) when new drug is prescribed.
Write **DATE** (DD/MM) that drug is administered in "Date" column (1 day per column = 7 days on this form).
Write **YOUR INITIALS** and the **TIME** the drug was administered in the relevant time rows (e.g. "SBO 13:00" in the "Afternoon" row). 24hr clock.
Record **DURATION** by cancelling the column after the number of days you would like the patient to receive the drug by writing **STOP**.

				**Date →**							
**Drug:**		**Start Date:**									
Route	*Dose*	*Freq*		Morning							
				Afternoon							
Name (print)		*Signature*		Evening							
				Night							
**Drug:**		**Start date:**									
Route	*Dose*	*Freq*		Morning							
				Afternoon							
Name (print)		*Signature*		Evening							
				Night							
**Drug:**		**Start Date:**									
Route	*Dose*	*Freq*		Morning							
				Afternoon							
Name (print)		*Signature*		Evening							
				Night							
**Drug:**		**Start Date:**									
Route	*Dose*	*Freq*		Morning							
				Afternoon							
Name (print)		*Signature*		Evening							
				Night							
				**Pharmacist Check**							

Adult Drug Chart v3.6 Patient name: ______________________

Patient ID: KT-☐-☐☐☐☐☐

Age___years Sex __

Weight___kg

Complete **DRUG INFORMATION** (route, dose, frequency, your name, signature) when new drug is prescribed.

Write **DATE** (DD/MM) that drug is administered in "Date" column (1 day per column = 7 days on this form).

Write **YOUR INITIALS** and the **TIME** the drug was administered in the relevant time rows. 24hr clock.

Record **DURATION** by cancelling the column after the number of days you would like the patient to receive the drug by writing **STOP**.

			**Date →**							
**Zinc Sulphate (20 mg)**		**Start Date:**								
Route<br>Oral	*Dose*<br>1 tablet	*Freq*<br>Once daily (for 10 days)	Morning							
			Afternoon							
Name (print)		*Signature*	Evening							
			Night							
**Multivitamin tablets**		**Start Date:**								
Route<br>Oral	*Dose*<br>1 tablet	*Freq*<br>Once daily	Morning							
			Afternoon							
Name (print)		*Signature*	Evening							
			Night							
**Paracetamol (500mg)**		**Start Date:**								
Route<br>Oral	*Dose*<br>2 tablets	*Freq*<br>4 times a day PRN	Morning							
			Afternoon							
Name (print)		*Signature*	Evening							
			Night							
		**Start Date:**								
Route	*Dose*	*Freq*	Morning							
			Afternoon							
Name (print)		*Signature*	Evening							
			Night							
			**Pharmacist Check**							

<u>Paediatric</u> Drug Chart v3.6 Patient name: ______________________ Patient ID: KT-☐-☐☐☐☐☐

Age___years Sex __
Weight___kg

Complete **DRUG INFORMATION** (route, dose, frequency, your name, signature) when new drug is prescribed.
Write **DATE** (DD/MM) that drug is <u>administered</u> in "Date" column (1 day per column = 7 days on this form).
Write **YOUR INITIALS** and the **TIME** the drug was <u>administered</u> in the relevant time rows. 24hr clock.
Record **DURATION** by cancelling the column after the number of days you would like the patient to receive the drug by writing **STOP**.

				**Date →**							
**Zinc Sulphate (20 mg)**	**Start Date:**										
Route Oral	*Dose*	*<12 months: ½ tablet* *> 12 months: 1 tablet*	*Freq* Once daily for 10 days	Morning							
				Afternoon							
Name (print)		*Signature*		Evening							
				Night							
**Multivitamin tablets**	**Start Date:**										
Route Oral		*Dose* 1 tablet	*Freq* Once daily	Morning							
				Afternoon							
Name (print)		*Signature*		Evening							
				Night							
**Paracetamol**	**Start Date:**										
Route Oral	*Dose*	*7-11 Kg: 120 mg* *12-17 Kg: 240 mg* *18-24 Kg: 360 mg* *25 - 40 Kg: 500 mg*	*Freq* Four times daily PRN	Morning							
				Afternoon							
Name (print)		*Signature*		Evening							
				Night							
**Vitamin A**	**Start Date:**										
Route Oral	*Dose*	*1- 6 months: 50,000 IU* *6-12 months: 100,000* *> 12 months: 200,000*	*Freq* Once daily for 2 days	Morning							
				Afternoon							
Name (print)		*Signature*		Evening							
				Night							
				**Pharmacist Check**							

Fluid infusion chart

The following chart was used by clinical staff to order and monitor intravenous fluids given to a patient.

SAVE THE CHILDREN – KERRY TOWN EBOLA TREATMENT CENTRE

INFUSION FLUIDS CHART (v3.4) **Patient name: ______________________________**

PATIENT ID: KT-☐-☐☐☐☐☐

**DATE**	**INFUSION FLUID**	**VOLUME**	**RATE**	**ROUTE**	Time	Given by	Time	Checked by	Time	Checked by	Time	Checked by

Lab request form

The following form was used by clinical staff to order lab tests from the 1) Public Health England on-site laboratory (for Ebola PCR and malaria rapid diagnostic tests) and 2) UK Ministry of Defense on-site laboratory (for various biochemistry blood tests).

LAB REQUEST FORM – SAVE THE CHILDREN – KERRY TOWN EBOLA TREATMENT CENTRE

DATE: ____ / ____ / 2015
DD / MM / YYYY

PATIENT ID #: KT–☐ –☐☐☐☐☐
KT-2 = Triage; KT-3 = Confirmed; KT-4 = morgue

BASIC PATIENT INFORMATION

Ward #: ______ **Bed #:**______

Name: Surname_______________________ Given names_______________________

Sex: ☐Male ☐Female

Age: _____ ☐ YEARS or ☐ MONTHS (for children under 1 year)

PRESCRIBER INFORMATION

Requested by (print your name): ____________________________________

Title: ____

LAB TESTS (PHE)

☐ **New admission (Malaria RDT + Ebola PCR)**

☐ **Repeat Ebola PCR**

LAB TESTS (MoD)

☐ **Full Blood Count (purple)**

☐ **Coagulation screen (blue)**

☐ **Amylyte 13 (green)**

OR

☐ **Metlac 12 (green)**

Special request

☐ **D-Dimer**

☐ **HIV**

☐ **Blood culture**

☐ **Dengue**

Amylyte 13
Sodium
Potassium
Urea
Creatinine
Glucose
Calcium
Albumin

Total Bilirubin
ALT
AST
CK
Amylase
CRP

Metlac 12
Sodium
Potassium
Urea
Creatinine
Glucose
Calcium
Albumin

Chloride
Magnesium
Lactate
Phosphate
Bicarbonate

Nutrition summary form

The following form was used by clinical staff daily to record the type of food a patient could consume (solid, semi-solid, liquid, nothing) for meal preparation by the kitchen.

NUTRITION SUMMARY – SAVE THE CHILDREN – KERRY TOWN EBOLA TREATMENT CENTRE

**DATE** (dd/mm/yyyy): ____ /____ / 2015	**WARD #:** _______

Fill in a line for each patient on the ward

Bed #	Patient ID #	Name	Sex	Age	Type of food patient can eat	Critically ill?	Comments (e.g. consumption issues)
1	KT-☐-☐☐☐☐☐				☐Solid ☐Semi-solid ☐Liquid ☐Nothing	☐Yes ☐No	
2	KT-☐-☐☐☐☐☐				☐Solid ☐Semi-solid ☐Liquid ☐Nothing	☐Yes ☐No	
3	KT-☐-☐☐☐☐☐				☐Solid ☐Semi-solid ☐Liquid ☐Nothing	☐Yes ☐No	
4	KT-☐-☐☐☐☐☐				☐Solid ☐Semi-solid ☐Liquid ☐Nothing	☐Yes ☐No	
5	KT-☐-☐☐☐☐☐				☐Solid ☐Semi-solid ☐Liquid ☐Nothing	☐Yes ☐No	
6	KT-☐-☐☐☐☐☐				☐Solid ☐Semi-solid ☐Liquid ☐Nothing	☐Yes ☐No	
7	KT-☐-☐☐☐☐☐				☐Solid ☐Semi-solid ☐Liquid ☐Nothing	☐Yes ☐No	
8	KT-☐-☐☐☐☐☐				☐Solid ☐Semi-solid ☐Liquid ☐Nothing	☐Yes ☐No	

Discharge forms

The following forms were completed by clinical staff when a patient was leaving the ward due to death, discharge home after recovery, or transfer to another facility. The first form (page 26) was deployed when the Kerry Town ETC in November 2014. The following two forms (pages 27 and 28) were deployed starting January 2015 when the suspect wards officially opened at the ETC.

DISCHARGE FORM – SAVE THE CHILDREN – KERRY TOWN EBOLA TREATMENT CENTRE

DATE: ____ / ____ / 2015
DD / MM / YYYY

PATIENT ID #: KT–☐ –☐☐☐☐☐
KT-2 = Triage; KT-3 = Confirmed; KT-4 = morgue

COMPLETE FORM IN WARD UPON DISCHARGE OR DEATH OF PATIENT

Final outcome: ☐ Deceased ☐ Discharged ☐ Transferred to other facility

***If Deceased*, date of death:** _____ /_____ / 2015
DD / MM / YYYY

If Discharged,

Discharge type: ☐ By staff ☐ Self-discharged ☐ Removed by family ☐Unknown

Did the patient have a confirmed negative test for Ebola? ☐YES ☐NO

If yes, ☐ never had Ebola (discharged from suspect ward)

OR

☐ recovered from Ebola (discharged from recovery ward)

Discharge medications provided?

If yes, list medications ____

If Transferred to other facility,

Reason for transfer: ____

Name of new facility: ____

District/town of new facility: ______________________________________

Discharge medications provided?

If yes, list medications ____

Form completed by (print name): ______________________________________

Signature: ____

Discharge form for Non-Ebola Patients from the ETC, Kerrytown

**Patient name & Age**		**CI number**	
**Admission date**		**Discharge date**	

PATIENT DID NOT MEET THE EBOLA CASE DEFINITION ☐

OR

PATIENT HAD A NEGATIVE EBOLA PCR AFTER > 3 DAYS OF SYMPTOMS ☐

This patient's symptoms began on the ________________ and the patient had a negative ebola PCR on the ________________

The patient was given a presumptive diagnosis of ____________________________

Discharge medication:

**Name of CHO/Dr:**			
**Signature:**		**Date:**	

ACCEPTING HOSPITAL:

**Patient name**		**Date of admission to ETC**	
**Hospital no.**		**Age**	

Date of symptom onset: _______________

Date of negative Ebola PCR test: _______________

Likely diagnosis:

Reason for referral:

Medications (with date started):

**Name of Dr/CHO:**			
**Signature:**		**Date:**	

Exit form

The following form was completed by staff when a patient was discharged from the facility. This form was for administrative purposes, including information for patient follow-up and provision of discharge packets.

EXIT FORM – SAVE THE CHILDREN – KERRY TOWN EBOLA TREATMENT CENTRE

DATE: ____ / ____ / 2015<br>DD / MM / YYYY	PATIENT ID #: KT–☐ –☐☐☐☐☐<br>KT-2 = Triage; KT-3 = Confirmed; KT-4 = morgue

COMPLETE FORM IN DISCHARGE TENT UPON DISCHARGE FROM ETC

Discharge pack given to: ☐ Patient ☐ Family of deceased patient

Name of patient: Surname____________________ **First name** ____________________

Name of recipient: Surname__________________ **First name** __________________

Mobile # __________________________ **2nd mobile #** __________________________

Where is the recipient going now: House#/Street (if any) ______________________

District _______________________ Chiefdom/Ward ______________________

Town/village ___________________

Mode of transportation: ☐Save the children vehicle ☐Taxi ☐Family ☐Other

Is the patient accompanied by SCI staff: ☐YES ☐NO

If yes, Name of the accompanying person: _______________________________

If no, reason : ____

Discharge package

Solidarity kit provided? ☐YES ☐NO

Hygiene kit provided? ☐YES ☐NO

Food ration provided? ☐YES ☐NO

Condoms provided? ☐YES ☐NO

Cash provided (Le 750,000)? ☐YES ☐NO

Certificate of discharge provided? ☐YES ☐NO

**Discharge package given by:**<br><br>**Signature:**	**Recipient name:**<br><br>**Signature:**

Evaluation form

The following form was used by staff to obtain feedback about the patient's experience during their stay at the Kerry Town Ebola treatment center. This form was not used for formal evaluation, and it was acknowledged that the answers would be biased because these forms were completed only by survivors on the day they were discharged from the treatment center.

EVALUATION FORM – SAVE THE CHILDREN – KERRY TOWN EBOLA TREATMENT CENTRE

DATE: ____ / ____ / 2015<br>DD / MM / YYYY	PATIENT ID #: KT–☐ –☐☐☐☐☐<br>KT-2 = Triage; KT-3 = Confirmed; KT-4 = morgue

COMPLETE FORM IN DISCHARGE TENT UPON DISCHARGE FROM ETC

Name of patient: Surname____________________ First name ____________________

For each of the topics below, please circle a number from 1 to 5 to describe your experience, where 1 is the **worst possible** and 5 is the **best possible**. If you have more comments to add, please write them in the box.

Please give your opinion of:

	Rating	Comments
The admission process	1 2 3 4 5	
Cleanliness of the ward	1 2 3 4 5	
The food	1 2 3 4 5	
The way staff behaved towards you	1 2 3 4 5	
Your medical care	1 2 3 4 5	
Your comfort	1 2 3 4 5	
Communication with your relatives	1 2 3 4 5	
Support after discharge	1 2 3 4 5	
The ETC overall	1 2 3 4 5	

Discharge certificate

The following form was completed by staff and provided to survivors upon discharge from the Kerry Town Ebola treatment center.

EBOLA TREATEMENT CENTER
KERRY TOWN

Save the Children

Government of Sierra Leone
Ministry of Health and Sanitation

CERTIFICATE OF DISCHARGE

We, hereby, certify that Mr/Mrs/Miss…………………………………………………............
has been successfully treated at the Kerry Town Ebola Treatment Centre and is now **free of Ebola**. He/she does not constitute any risk to the community in any way.

Kerry Town, Ebola Treatment Center

Date :

Clinical Lead , Name and signature :

UKaid from the British people

Death certificate

The following form was completed by staff for patients who died during their stay at the Kerry Town Ebola treatment center.

Government of Sierra Leone
Ministry of Health and Sanitation

Save the Children

DEATH CERTIFICATE

KERRY TOWN – EBOLA TREATMENT CENTER

We, hereby, certify that Mr/Mrs/Miss ………………………………………………………………,

admitted in the Ebola Treatment Center at Kerry Town on ………/………/………,

died in our facility. He was tested as an Ebola positive patient.

Date of death: …. /…./…. Time of death: ………

Name and Signature

Clinical Doctor
